# Comparative Analysis of Chromosome Repeat DNA Patterns in Four *Amaranthus* Species

**DOI:** 10.3390/ijms262211026

**Published:** 2025-11-14

**Authors:** Alexandra V. Amosova, Olga Yu. Yurkevich, Alexey R. Semenov, Murat S. Gins, Julia V. Kalnyuk, Lyudmila V. Zemtsova, Alexander I. Morozov, Ekaterina D. Badaeva, Svyatoslav A. Zoshchuk, Olga V. Muravenko

**Affiliations:** 1 Engelhardt Institute of Molecular Biology, Russian Academy of Sciences, 119991 Moscow, Russia; 2 Federal State Budgetary Scientific Institution, Federal Scientific Center for Vegetable Growing, 143080 Odintsovo, Russia; 3 All-Russian Institute of Medicinal and Aromatic Plants, Federal Agency for Scientific Organizations, 117216 Moscow, Russia

**Keywords:** *Amaranthus* L., repeatome, FISH, molecular cytogenetic markers, karyotype, chromosome diversity

## Abstract

*Amaranthus* L. includes valuable and promising crops of multi-purpose use, having high morphological diversity and complicated taxonomy. Their karyotypes and genomic relationships remain insufficiently studied. For the first time, a comparative repeatome analysis of *Amaranthus tricolor* L., *Amaranthus cruentus* L., and *Amaranthus hypochondriacus* L. was performed based on the high-throughput sequencing data obtained via bioinformatic analyses using the RepeatExplorer2/TAREAN/DANTE_LTR pipelines. Interspecific variations in the abundance of Ty1 Copia and Ty3 Gypsy retroelements, DNA transposons, and ribosomal and satellite DNA (satDNA) were detected. Based on fluorescence *in situ* hybridization (FISH), chromosome mapping of 45S rDNA, 5S rDNA, and satDNAs AmC9 and AmC70, and unique karyograms of *A. tricolor*, *A. cruentus*, *Amaranthus paniculatus* L., and *A. hypochondriacus* were constructed. The analysis of the interspecies genome diversity/similarity in DNA repeat contents, sequences of the identified satDNAs, and chromosome distribution patterns of the studied molecular markers indicated that these species might also share a common evolutionary ancestor. However, the genomes of *A. cruentus*, *A. paniculatus*, and *A. hypochondriacus* were more similar compared to *A. tricolor*, which aligns with the previous phylogenetic data. Our results demonstrate that cytogenomic studies might provide important data on *Amaranthus* species relationships elucidating taxonomy and evolution of these valuable crops.

## 1. Introduction

*Amaranthus* L. (*Amaranthaceae* Juss.) is a widespread genus of predominantly annual herbaceous plants. This genus includes about 100 species native to warm and temperate regions [1]. The *Amaranthus* species adapt easily to unfavorable environmental conditions. They have increased phenotypic plasticity and genetic diversity, and C4 photosynthesis enables them to have a high photosynthetic rate [1,2]. Currently, amaranths (the common name for plants in the genus *Amaranthus*) are used as promising donors of useful genes associated with abiotic stress resistance in plants [3,4,5].

Several species of this genus, including *Amaranthus caudatus* L., *Amaranthus cruentus* L., *Amaranthus paniculatus* L., *Amaranthus hypochondriacus* L., *Amaranthus tricolor* L., *Amaranthus hybridus* L., *Amaranthus gangeticus* L., *Amaranthus blitum* L., *Amaranthus viridis* L., *Amaranthus spinosus* L., *Amaranthus muricatus* (Gillies ex Moq.) Hieron., *Amaranthus dubius* Mart. ex Thell., and *Amaranthus tunetanus* Iamonico and El Mokni., are economically important domesticated species [6,7]. The *Amaranthus* leaves, roots, and seeds (grains) contain high protein content and a number of beneficial bioactive compounds, including antioxidants (polyphenols, squalene, α-tocopherol), important macro- and microelements (iron, calcium, magnesium, zinc), vitamins, essential amino acids, phytochemicals, lectins, anthocyanins, and bioactive peptides [1,8,9,10,11]. They are promising high-yield crops which can be used for human consumption (pseudocereals, leafy vegetables); animal feed; prevention and treatment of many viral and inflammatory diseases, metabolic and cardiac disorders, diseases of the digestive system, and burns; and other nutraceutical purposes [6,12,13,14,15,16]. Some *Amaranthus* species, including *A. cruentus*, *A. caudatus*, *A. hypochondricus*, *A. paniculatus* and *A. tricolor*, are used as ornamentals due to their brightly colored leaves and unusually shaped flowers with spike-like panicles and numerous small blossoms [1].

The taxonomy of the genus *Amaranthus* is complicated by its wide geographical distribution, the high levels of phenotypic variability of its representatives, and the presence of various intraspecific hybrids [1,17,18,19]. This could result in violations of nomenclature and incorrect use of species names [1,20]. Several taxonomic revisions of this genus were reported [21,22,23,24,25]. The most commonly used intrageneric classification of *Amaranthus* is based mainly on the morphological characters of inflorescences, flowers, fruit dehiscence mode, and sexual characteristics [22]. This classification recognized three subgenera—*Acnida* L. Aellen ex K.R. Robertson, *Albersia* Kunth Gren. & Godr., and *Amaranthus*—with further differentiation into sections and subsections within each subgenus. At the same time, the relationships among these subgenera remain unclear, and species delimitation is often ambiguous due to interspecific hybridization events and high phenotypic plasticity [1,22,25]. In particular, three economically important species—*A. cruentus*, *A. paniculatus*, and *A. hypochondriacus*—belong to the subgenus *Amaranthus* sect. *Amaranthus* subsect. *Hybrida* Mosyakin & K. R. Robertson that involves a group of polymorphic and hybrid forms [22]. The species *A. paniculatus* is also considered to be a synonym of *A. cruentus* or *A. hybridus* var. *paniculatus* L. [25]. The economically important *A. tricolor* belongs to the subgenus *Albersia* sect. *Pyxidium* Moq. that includes a diverse group of highly polymorphic species.

Recent molecular studies [26,27,28,29,30,31] highlighted that the classification of Mosyakin & Robertson [22] does not always match the clades identified in the phylogenetic trees. Based on the analysis of restriction site variations in the nuclear and cytoplasmic DNA of *A. caudatus* and *A. cruentus*, these species were more closely related to each other than to *A. hypochondriacus* [32]. However, phylogenetic evaluation of genetic diversity using competitive allele SNPs indicated that *A. cruentus* and *A. hypochondriacus* were related and more distant from *A. caudatus* [30]. Molecular studies based on nuclear internal transcribed spacers (ITSs) and chloroplast DNA (matK, trnL) recognized five groups within the genus *Amaranthus* (Dioecious/Pumilus clade; Hybridus clade; Galapagos (three clades); Eurasian + South African + Australian (ESA) clade; ESA + South American clade) [31]. Phylogenic studies of the chloroplast genomes of 27 *Amaranthus* species across all the three subgenera have confirmed the monophyly of the subgenus *Amaranthus* but found *Acnida* and *Alberisa* to be paraphyletic [33]. Based on the whole genome sequencing data and a genome-wide assembly in *A. hypochondriacus*, *A. cruentus*, and *A. tricolor*, it was shown that the ancestors of the genus *Amaranthus* shared a WGD (Whole Genome Duplication) event that occurred approximately 26.56 Mya [34,35,36]. The subgenera *Albersia* (which includes *A. tricolor*) diverged from the last common ancestor of subgenera *Amaranthus* and *Acnida* 5.73 Mya, and the subgenus *Amaranthus* diverged from *Acnida* 1.72 Mya [35]. It was also reported that *A. cruentus* and *A. hypochondriacus* (both belong to the subgenus *Amaranthus*) diverged from each other approximately 1.45 Mya [34].

Three basic chromosome numbers (*n* = 14, 16, and 17) were revealed within the genus [37,38,39,40]. In some *Amaranthus* species (e.g., in *A. caudatus*, *A. cruentus*, and *A. hybridus*), intraspecific variability in chromosome numbers was detected [41]. Most species were diploid except for one tetraploid species, *A. dubius* (2*n* = 4*x* = 64) [40]. The karyotypes of amaranths contained small-sized (0.8–3.5 μm) chromosomes [42,43,44], which made a cytogenetic analysis rather difficult, and currently, only a few molecular cytogenetic studies have been reported [41,45,46,47]. Depending on the *Amaranthus* species, 1–3 chromosome pairs bearing 45S rDNA clusters and 1–6 pairs with 5S rDNA loci were revealed using FISH (fluorescence in situ hybridization). In addition, intra- and interspecies variability in rDNA clusters was detected [41,47].

The aim of the present study was to perform a comparative bioinformatic analysis of repeatomes of the valuable species *A. tricolor*, *A. cruentus*, and *A. hypochondriacus* belonging to two related subgenera, *Albersia* and *Amaranthus*, to elucidate the similarity/diversity in the DNA repeat content in their genomes. The bioinformatic analysis included an examination of the available WGS (whole genome sequencing) data using RepeatExplorer2/TAREAN/DANTE_LTR pipelines as well as BLAST (Basic Local Alignment Search Tool). Based on a FISH mapping of 45S rDNA, 5S rDNA together with two common identified satellite DNA families (satDNAs), we also carried out a comparative characterization of the karyotypes of five varieties of these species (*A. cruentus* var. Dyuimovochka, *A. hypochondriacus* var. Kizlyarets, *A hypochondriacus* var. Krepysh, *A. paniculatus* var. Fakel, and *A. tricolor* var. Valentina) to study chromosome diversity and detect possible aberrations, which is important for breeding.

## 2. Results

### 2.1. Comparative Analyses of DNA Repeats Identified in Genomes of A. tricolor, A. cruentus, and A. hypochondriacus

For the first time, we carried out a comparative analysis of the repeatomes of *A. tricolor*, *A. cruentus*, and *A. hypochondriacus* based on the content of transposable elements (TEs) and major satDNA families identified in their genomes. A comparative analysis of the DNA Repeats identified in the genomes of these species demonstrated that retrotransposons (Class I) were most abundant elements (Supplementary Table S1). In the repeatome of *A. tricolor*, the largest proportion of these retroelements (14.41%), including Ty1 Copia (7.19%) and Ty3 Gypsy (5.83%), was revealed when compared with *A. cruentus* (11.06%, 4.28%, and 5.37%, respectively) and *A. hypochondriacus* (10.07%, 4.00%, and 3.06%, respectively).

**Figure 1:**
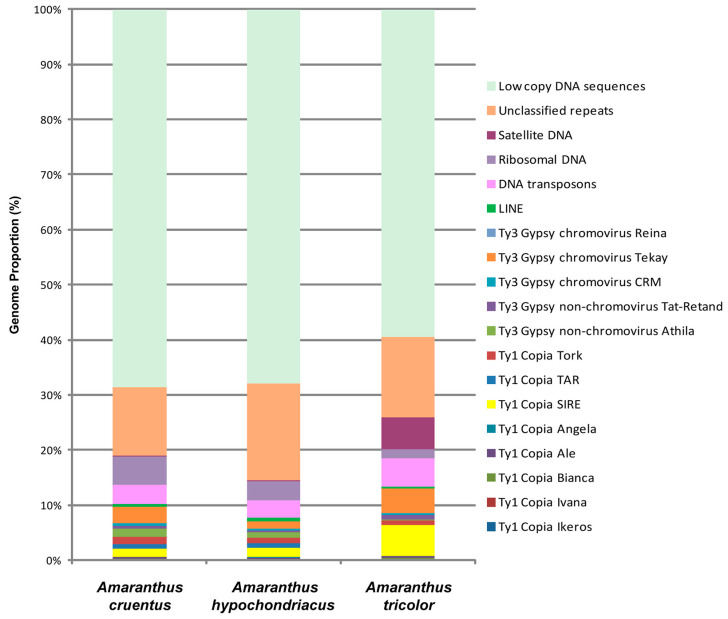
Genome proportions of the abundant DNA repeats identified in *Amaranthus tricolor* (green), *Amaranthus cruentus* (orange), and *Amaranthus hypochondriacus* (brown). Each proportion was calculated using RepeatExplorer2 as a ratio of the number of reads specific to a particular repeat type to the sum of all reads used in the cluster analysis.

In *A. cruentus* (unlike *A. tricolor* and *A. hypochondriacus*), Ty3 Gypsy retroelements were more abundant than Ty1 Copia retrotransposons. In *A. tricolor*, the proportion of SIRE (Ty1 Copia superfamily) significantly exceeded the proportions revealed in the other two species. At the same time, TAR and Tork retroelements were more abundant in *A. cruentus* and *A. hypochondriacus* than in *A. tricolor* (Figure 1, Supplementary Table S1*).*

In the Ty3 Gypsy superfamily, chromovirus Tekay and non-chromovirus Athila were most abundant, although among *A. tricolor*, *A. cruentus*, and *A. hypochondriacus*, differences in content of non-chromovirus Athila (0.15%, 1.44%, and 1.07%, respectively) and chromovirus Tekay (4.50%, 2.90%, and 1.30%, respectively) were also observed (Figure 1, Supplementary Table S1).

In *A. tricolor*, *A. cruentus*, and *A. hypochondriacus*, different proportions of DNA transposons (Class II) (5.04%, 3.52%, and 3.07%, respectively) were revealed. In DNA transposons, CACTA (1.73%, 0.82%, and 1.24%, respectively) and MuDR_Mutator (1.99%, 1.46%, and 0.77%, respectively) were most abundant.

In *A. tricolor*, *A. cruentus*, and *A. hypochondriacus*, differences in proportions of ribosomal DNA (1.72%, 5.06%, 3.54%, respectively) and satellite DNA (5.70%, 0.27%, and 0.16%, respectively) were revealed (Figure 1, Supplementary Table S1). Moreover, the TAREAN/DANTE_LTR pipelines identified different numbers of putative tandem DNA repeats in the genomes of *A. tricolor* (AmT repeats), *A. cruentus* (AmC repeats), and *A. hypochondriacus* (AmH repeats). In the genome of *A. cruentus*, the highest number of tandem repeats (seven high-confidence and four low-confidence putative satDNAs, as well as two LTR repeats) was revealed. In *A. tricolor*, the lowest number of repeats (four high-confidence and two low-confidence putative satDNAs, as well as one LTR repeat) was detected. In the genome of *A. hypochondricus*, two high-confidence and five low-confidence satDNAs, as well as three LTR repeats were found (Supplementary Tables S2–S13).

Generally, the obtained results show that, despite the identified differences in the content and composition of TEs and major DNA repeat families in the repeatomes of the studied species, *A. cruentus* and *A. hypochondriacus* were more similar compared to *A. tricolor.*

### 2.2. BLAST Analysis of the Identified Tandem DNAs

**Figure 2:**
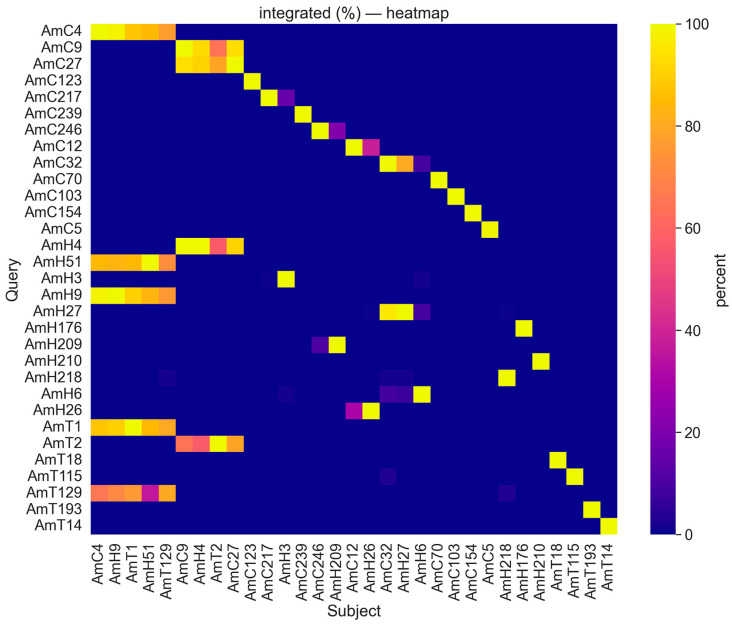
The heatmap constructed according to BLAST results (identity/coverage) demonstrating sequence similarity among the tandem DNA repeats identified in the genomes of *Amaranthus tricolor*, *Amaranthus cruentus*, and *Amaranthus hypochondriacus*. The percentage of similarity between matching repeated sequences is shown in different colors. The color scale is indicated on the right.

The BLAST analysis revealed high intraspecific sequence similarity between satDNAs identified in the genomes of *A. cruentus* (AmC9 and AmC27, 100%/93% of coverage/identity), *A. hypochondriacus* (AmH9 and AmH51, 98%/85% of coverage/identity), and *A. tricolor* (AmT1 and AmT129, 100%/81% of coverage/identity) (Figure 2, Supplementary Figure S1, Supplementary Tables S2–S13).

High interspecies sequence similarity was detected between several repeats of the three studied species: AmT1 and repeats AmC4 (96%/91% of coverage/identity), AmH51 (100%/84% of coverage/identity), and AmH9 (100%/89% of coverage/identity); AmT2 and repeats AmC27 (86%/92% of coverage/identity), AmC9 (67%/96% of coverage/identity), and AmH51 (60%/96% of coverage/identity); AmT129 and repeats AmC4 (85%/75% of coverage/identity), AmH9 (92%/78% of coverage/identity), and AmH51 (49%/72% of coverage/identity) (Figure 2, Supplementary Tables S2–S14).

Moreover, high sequence similarity was revealed between several abundant repeats of *A. cruentus* and *A. hypochondriacus*: AmC4 and both repeats AmH51 (99%/85% of coverage/identity) and AmH9 (100%/98% of coverage/identity); AmC9 and AmH4 (93%/100% of coverage/identity); AmC27 and AmH4 (100%/91% of coverage/identity); AmC32 and AmH27 (83%/97% of coverage/identity); LTR AmC12 and AmH26 (41%/93% of coverage/identity) (Figure 2, Supplementary Tables S2–S14).

In addition, tandem DNAs AmC32, AmH27, and LTR AmC5 demonstrated sequence similarity with *Amaranthus palmeri*; and AmH210 showed sequence similarity with *Amaranthus retroflexus.* According to BLAST, intra- or interspecific satDNA sequence similarity was not revealed for several identified tandem DNAs, including AmT18, AmT115, AmT193, LTR AmT14, AmC239, AmC32, AmC103, AmC154, AmC70, AmC154, AmH176, AmH209, and AmH218 (Figure 2, Supplementary Tables S2–S13).

Thus, the bioinformatic analysis revealed high intra- and interspecies sequence similarity of major DNA repeat families between the studied species. Seven satDNAs identified in the genome of *A. cruentus* demonstrated sequence identity with nine satDNAs of *A. hypochondriacus.* At the same time, three satDNAs of *A. tricolor* had sequence identity with three satDNAs of *A. cruentus* and two satDNAs of *A. hypochondriacus.*

### 2.3. Structural Variations in Chromosomes Revealed by FISH

For the first time, comparative chromosome analysis was carried out in five *Amaranthus* varieties and one *A. cruentus* specimen: *A. cruentus* var. Dyuimovochka, *A. cruentus* (accession number 842-04), *A. hypochondriacus* var. Kizlyarets, *A. hypochondriacus* var. Krepysh, *A. paniculatus* var. Fakel, and *A. tricolor* var. Valentina.

**Figure 3:**
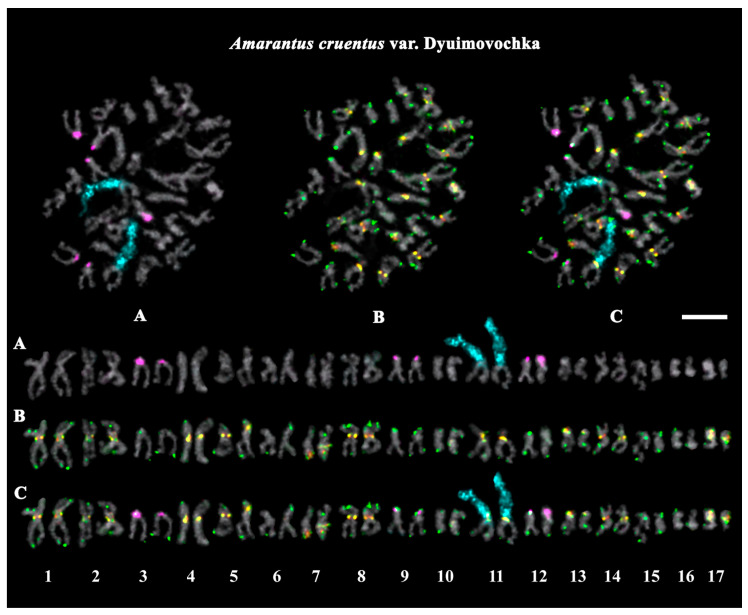
FISH-based localization of (**A**) 45S rDNA (aqua) and 5S rDNA (purple); (**B**) AmC9 (red) and AmC70 (green); and (**C**) 45S rDNA (aqua), 5S rDNA (purple), AmC9 (red), and AmC70 (green) on chromosomes of *Amaranthus cruentus* var. Dyuimovochka. DAPI-staining—gray. Co-localization of AmC9 and AmC70—yellow. Scale bar—5 μm.

**Figure 4:**
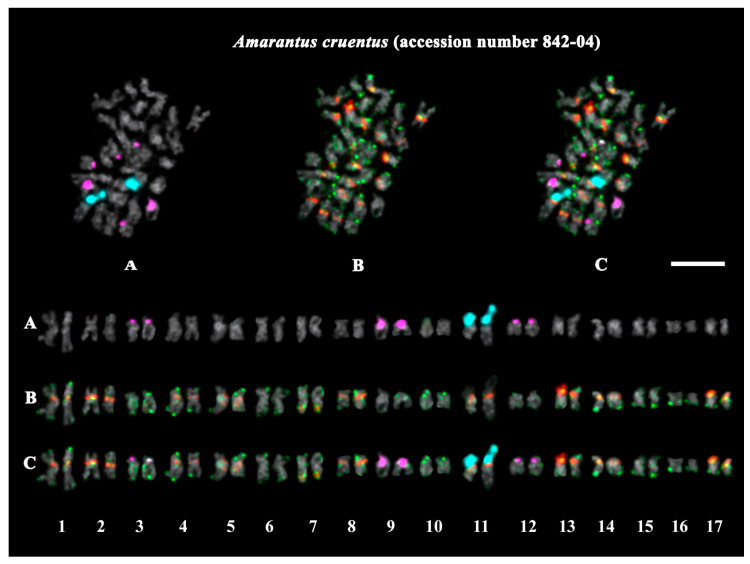
FISH-based localization of (**A**) 45S rDNA (aqua) and 5S rDNA (purple); (**B**) AmC9 (red) and AmC70 (green); and (**C**) 45S rDNA (aqua), 5S rDNA (purple), AmC9 (red), and AmC70 (green) on chromosomes of the specimen of *Amaranthus cruentus* (accession number 842-04). DAPI-staining—gray. Co-localization of AmC9 and AmC70—yellow. Scale bar—5 μm.

**Figure 5:**
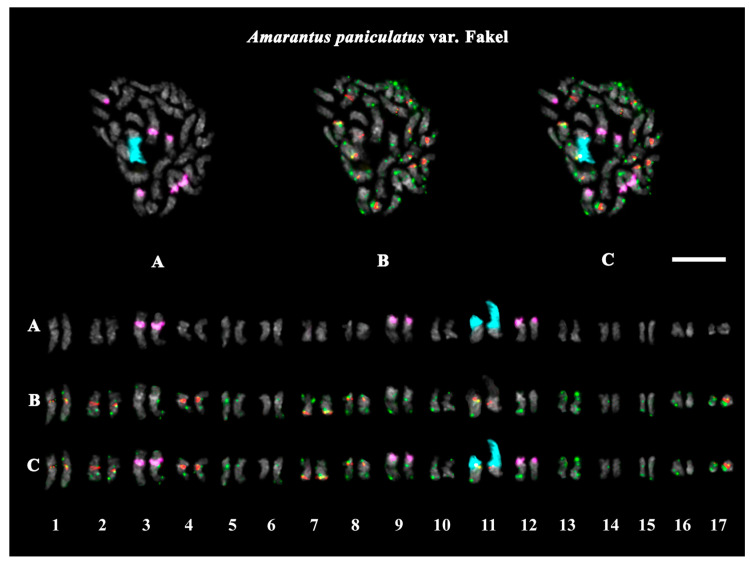
FISH-based localization of (**A**) 45S rDNA (aqua) and 5S rDNA (purple); (**B**) AmC9 (red) and AmC70 (green); and (**C**) 45S rDNA (aqua), 5S rDNA (purple), AmC9 (red), and AmC70 (green) on chromosomes of *Amaranthus paniculatus* var. Fakel. DAPI-staining—gray. Co-localization of AmC9 and AmC70—yellow. Scale bar—5 μm.

**Figure 6:**
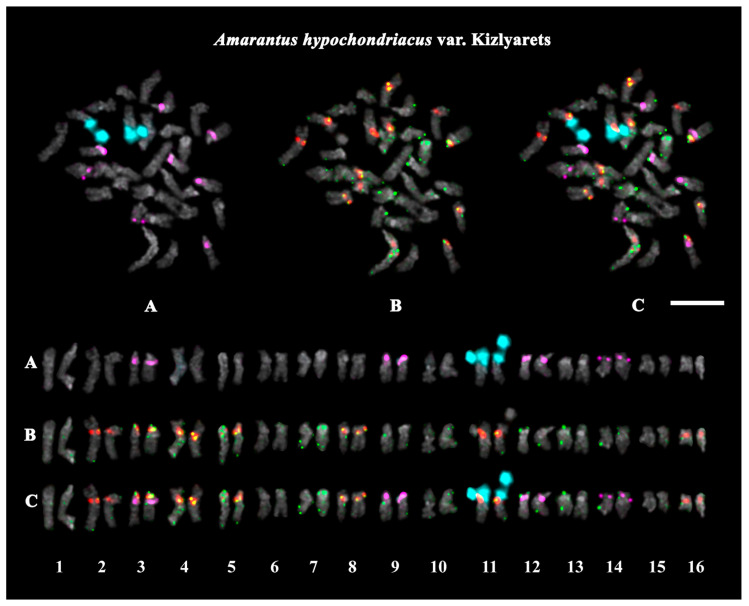
FISH-based localization of (**A**) 45S rDNA (aqua) and 5S rDNA (purple); (**B**) AmC9 (red) and AmC70 (green); and (**C**) 45S rDNA (aqua), 5S rDNA (purple), AmC9 (red), and AmC70 (green) on chromosomes of *Amaranthus hypochondriacus* var. Kizlyarets. DAPI-staining—gray. Co-localization of AmC9 and AmC70—yellow. Scale bar—5 μm.

**Figure 7:**
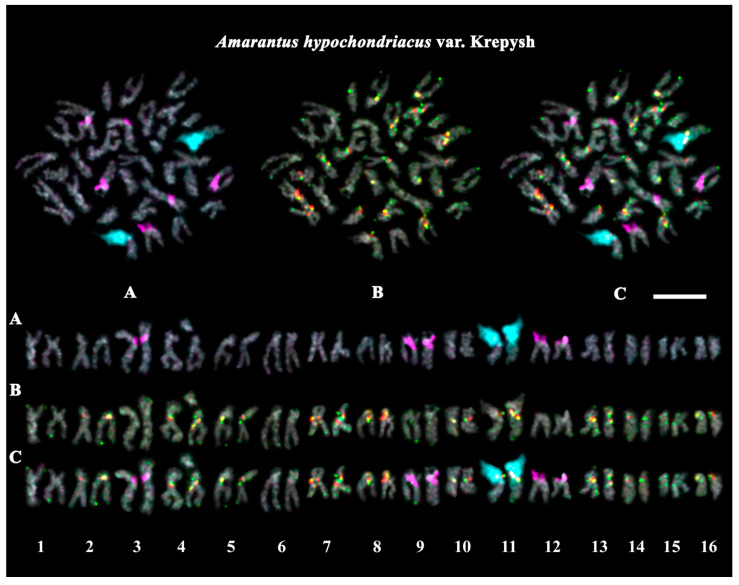
FISH-based localization of (**A**) 45S rDNA (aqua) and 5S rDNA (purple); (**B**) AmC9 (red) and AmC70 (green); and (**C**) 45S rDNA (aqua), 5S rDNA (purple), AmC9 (red), and AmC70 (green) on chromosomes of *Amaranthus hypochondriacus* var. Krepysh. DAPI-staining—gray. Co-localization of AmC9 and AmC70—yellow. Scale bar—5 μm.

**Figure 8:**
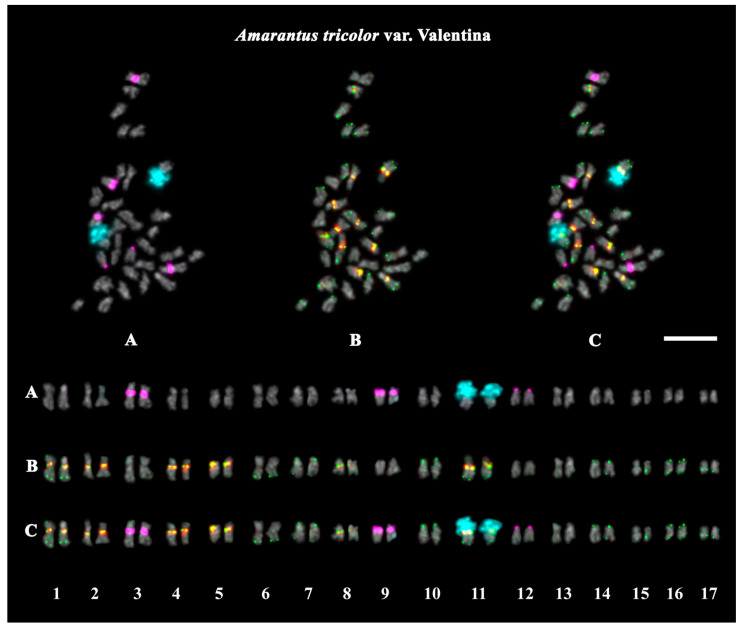
FISH-based localization of (**A**) 45S rDNA (aqua) and 5S rDNA (purple); (**B**) AmC9 (red) and AmC70 (green); and (**C**) 45S rDNA (aqua), 5S rDNA (purple), AmC9 (red), and AmC70 (green) on chromosomes of *Amaranthus tricolor* var. Valentina. DAPI-staining—gray. Co-localization of AmC9 and AmC70—yellow. Scale bar—5 μm.

The studied accessions of *A. cruentus*, *A. paniculatus*, and *A. tricolor* presented diploid karyotypes with 2*n* = 2*x* = 34 chromosomes. In the karyotypes of both *A. hypochondriacus* varieties, 2*n* = 2*x* = 32 chromosomes were observed (Figure 3, Figure 4, Figure 5, Figure 6, Figure 7 and Figure 8).

FISH revealed large 45S rDNA clusters in the terminal regions of the short arms of chromosome pair 11, and this chromosomal position of 45S rDNA sites was conservative for all studied *Amaranthus* samples (Figure 3A, Figure 4A, Figure 5A, Figure 6A, Figure 7A and Figure 8A). Clusters of 5S rDNA were observed on chromosome pairs 3 (in the proximal region of the short arms), 9 (in the distal regions of the short arms), and 12 (in the distal regions of the short arms) in all studied accessions (Figure 3A, Figure 4A, Figure 5A, Figure 6A, Figure 7A and Figure 8A). In the karyotype of *A. hypochondriacus* var. Kizlyarets, clusters of 5S rDNA were also revealed in the distal regions of the short arms of chromosome pair 14 (Figure 6A).

Depending on the *Amaranthus* species, clusters of AmC9 were localized on 6–11 chromosome pairs. Particularly, in *A. tricolor*, AmC9 clusters were observed in the pericentromeric regions of chromosome pairs 1, 2, 4, 5, 8, and 11. In *A. cruentus*, signals of this oligonucleotide probe were visualized in the pericentromeric regions of chromosome pairs 1, 2, 4, 5, 8, 11, and 14; in the distal regions of the long arms of chromosome pair 7 (a double cluster); as well as in the proximal regions of the short arms of chromosome pairs 13 and 17. In *A. paniculatus*, AmC9 signals were observed in the pericentromeric regions of chromosome pairs 1, 2, 4, 8, and 11; in the distal regions of the long arms of chromosome pair 7 (a single cluster); as well as in the proximal regions of the short arms of chromosome pair 17. In *A. hypochondriacus*, AmC9 signals were detected in the pericentromeric regions of chromosome pairs 2, 4, 7 (polymorphic), 8, 10 (polymorphic), 11, and 13 (polymorphic); in the distal regions of the short arms of chromosome pairs 3 and 14 (polymorphic); and in the proximal regions of the short arms of chromosome pairs 5 and 17 (Figure 3B, Figure 4B, Figure 5B, Figure 6B, Figure 7B and Figure 8B). Clusters of AmC70 were revealed in the pericentromeric and/or terminal regions of most chromosomes of the studied accessions (Figure 3B, Figure 4B, Figure 5B, Figure 6B, Figure 7B and Figure 8B).

The chromosome analysis of the studied *Amaranthus* varieties and the *A. cruentus* specimen (accession number 842-04) did not reveal significant chromosome rearrangements in their karyotypes.

**Figure 9:**
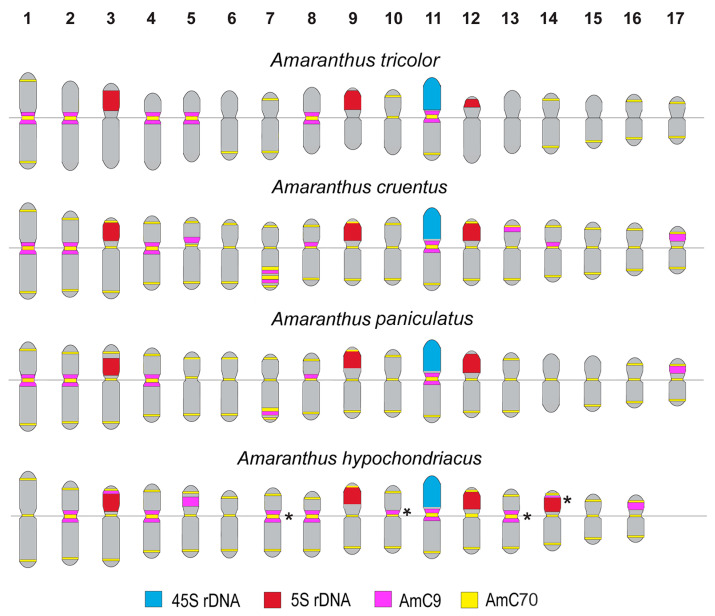
FISH-based idiogram schemes demonstrating the positions of sites of 45S rDNA (blue), 5S rDNA (red), AmC9 (yellow), and AmC70 (purple) on chromosomes of the studied *Amaranthus* species. Asterisks indicate the polymorphic sites.

Based on patterns of chromosome distribution of 45S rDNA, 5S rDNA, AmC9, and AmC70 in the karyotypes of the studied *Amaranthus* species, the generalized idiogram-schemes were constructed (Figure 9).

Thus, FISH-based mapping of two common satDNAs in a combination with 45S and 5S rDNA repeats on chromosomes of six *Amaranthus* accessions (including four species/five varieties) showed their species-specific chromosome distribution on chromosomes of the studied amarants. Using this approach, we identified homologous chromosome pairs in karyotypes and constructed the karyograms and species idiogramschemes demonstrating the chromosome positions of the studied molecular markers.

## 3. Discussion

Repetitive DNAs make up a significant portion of plant genomes. These DNA sequences undergo evolutionary changes and can be used as genetic markers to analyze genetic diversity and relationships within and between different species [48]. Among the studied *Amaranthus* species, we observed interspecies variations in genome proportions of TEs. These mobile repetitive DNAs can be copied and moved to new locations in the genome, contributing significantly to genome evolution [49]. Depending on the mechanism of transposition, TEs are divided into two main types: RNA transposons or retrotransposons (Class I) and DNA transposons (Class II) [49]. In plants, the abundance of TEs constitutes up to 90% of the genome [50]. In *A. tricolor*, we observed the largest proportions of LTR retrotransposons (14.41%) and DNA transposons (5.04%) compared with *A. cruentus* (11.06% and 3.52%, respectively) and *A. hypochondriacus* (10.07% and 3.07%, respectively), with the most significant interspecies differences in abundance of the Ty1 Copia SIRE, Ty3 Gypsy chromovirus Tekay, CACTA, and MuDR_Mutator lineages. These data are consistent with the genome sizes of *A. tricolor* (approximately 520 Mb), *A. cruentus* (370.9–399 Mb), and *A. hypochondriacus* (404–466 Mb) [34,35,51]. Genome size is an intrinsic property of plants, and intra- and interspecific variations in genome size may reflect changes occurring during speciation [52]. LTR retrotransposons include two major superfamilies, Ty1 Copia and Ty3 Gypsy, and several specific subfamilies. These retroelements can contribute to the genome size variations in plants [52,53,54]. They can replicate via a ‘copy and paste’ mechanism increasing the genome size. At the same time, they can reduce the genome size through both accumulation of deletions and/or solo LTR formation [52,54,55]. As previously reported, some changes in plant genomes occurred during speciation and were correlated with variations in the content of the SIRE, Athila (Ty3 Gypsy), and CACTA families [56]. DNA transposons also influence genome size. In particular, they can ‘cut and paste’ themselves into new locations within the genome, which leads to genome expansion, and the proportion of DNA transposons within a genome correlates with overall genome size variation [50]. Our results are generally consistent with the data obtained previously for other samples of these *Amaranthus* species [34,56].

Ribosomal DNA (rDNA) makes up a substantial repetitive fraction of a plant genome. The rDNA families are organized into multiple tandem repeats of the 45S rRNA genes (encoding 18S, 5.8S, and 25S rRNAs) and 5S rRNA genes, which are involved in the processes of protein synthesis [57]. In the present study, interspecies differences in genome proportions of ribosomal DNA were revealed, which may be related to the processes occurring in *Amaranthus* genomes during speciation. As previously reported, rDNA loci are the predominant sites of repeated recombination [58], which may cause both intragenomic variations in rDNA copy number and the amplification of new arrays [59]. Moreover, rDNA arrays and neighboring regions can be the targets for insertions of transposable elements [60].

Satellite DNA forms long tandem arrays of repeated DNA units which may accumulate changes in copy number and sequence over time, contributing to variability in genome structure and speciation [48,52,61]. Many satDNAs undergo rapid evolution, exhibiting high interspecific variability in sequence and chromosomal distribution due to rapid homogenization and turnover rates. This might result in the appearance of species-specific satDNAs and divergent evolutionary patterns. Such satDNAs can be used as taxonomic markers for closely related species [35,47]. The sequences of other satDNAs may be highly conserved, demonstrating different evolutionary patterns even within the same genus [62]. A significant fraction of satDNAs demonstrate sequence similarity between closely related species, indicating their common evolutionary history [63,64]. Comparative repeatome analysis helps to identify the common origin or divergence, clarifying the phylogenetic relationships in plant species. Between *A. tricolor* and both *A. cruentus* and *A. hypochondriacu*, we revealed interspecies differences in the genome proportions of several DNA repeats, including satDNAs, as well as in the numbers of putative satDNA families. However, several satDNAs were common to the three studied *Amaranthus* species (AmT1, AmT129, AmC4, AmH9, and AmH51, as well as, AmT2, AmC9, AmC27, and AmH51). At the same time, more similarities were observed between *A. cruentus* and *A. hypochondriacus* compared to *A. tricolor*. BLAST analysis detected high sequence similarity between the most abundant tandem DNA repeats identified in the genomes of *A. cruentus* and *A. hypochondriacus*, confirming their close relationships. Our results are consistent with the previous phylogenetic data, which placed *A. tricolor* into the subgenus *Albersia*, and both *A. cruentus* and *A. hypochondriacus* were assigned to the subgenus *Amaranthus* [22,35]. Moreover, sequence similarities were revealed between some tandem DNA repeats identified in the genomes of *A. cruentus* and *A. hypochondriacus* and repeats of the weeds *A. palmeri* and *A. retroflexus*, which are assigned to the subgenus *Acnida* [22]. These results indicate that they might also share a common evolutionary ancestor.

In the present study, molecular cytogenetic analysis was carried out in six *Amaranthus* accessions, including four species and five varieties. In the studied samples, we observed constant basic chromosome numbers: *n* = 16 (for both *A. hypochondriacus* accessions), and *n* = 17 (for both *A. cruentus* accessions, *A. paniculatus*, and *A. tricolor*). These chromosome numbers were consistent with those available at the Index to Plant Chromosome Number (www.tropicos.org/Project/IPCN, accessed on 20 September 2025) as well as the chromosome numbers reported earlier for other accessions [37,38,41,45,47]. At the same time, variability in chromosome numbers (both *n* = 16 and 17) was previously reported for several *Amaranthus* species belonging to the subgenus *Amaranthus*, including *A. caudatus*, *A. cruentus*, and *A. hybridus* [41], which could be related to the peculiarities of local samples (e.g., their hybrid nature) and/or very small sizes of chromosomes, which complicate chromosomal analysis. In our study, using a DNA intercalator (α-monobromonaphthalene) allowed us to enlarge chromosome sizes in the karyotypes of the amaranths and increase the resolution of chromosomal analysis, which was necessary to conduct FISH mapping of four molecular markers on chromosomes.

Ribosomal DNAs are useful cytogenetic markers for chromosome analysis. The clusters of 45S rDNA and 5S rDNA are usually localized in GC-rich heterochromatic regions and often used as chromosome markers in FISH-based assays due to their relatively conserved nature and abundance as ‘house-keeping genes’ [65]. FISH-based patterns of their chromosome distribution provide important information for elucidating genome organization and chromosomal relationships between plant taxa [66,67]. The ribosomal DNAs, as chromosome markers, are widely used for discovering genome restructuring, intra- and interspecies genetic diversity, and re-discovering the status of plant species [68,69,70,71].

In the present study, FISH analysis revealed one pair of chromosomes bearing large clusters of 45S rDNA in the karyotypes of all studied *Amaranthus* samples, and the chromosomal position of the 45S rDNA sites was conservative. These results confirm the diploid nature of the studied *Amaranthus* samples and agrees with the previous reports [41,46,47].

The loci of 5S rDNA were localized in the terminal regions of different chromosomes. We detected three pairs of chromosomes with 5S rDNA in the karyotypes of *A. tricolor*, *A. cruentus*, and *A. paniculatus*, and four 5S rDNA loci in the var. Kizlyarets (*A. hypochondriacus*). These results mainly agreed with the previous reports [41,47], although variability in the number of 5S rDNA was earlier revealed in *A. cruentus* [41]. Moreover, we detected only three chromosome pairs with 5S rDNA in the karyotype of the other variety of *A. hypochondriacus* (var. Krepysh), which demonstrated, for the first time, the presence of intraspecies differences in number of 5S rDNA sites in this species. Intra- and interspecies variations in number and location of rDNA sites could indicate their mobility in the recent evolutionary history of the species [41]. It was previously suggested that changes in the number of rDNA sites might result from chromosome rearrangements, amplification or deletion of the rDNA copy number during both homologous and nonhomologous unequal crossing over, and a transposon-mediated transposition of sequences containing rDNA repeats [72,73].

Clusters of satDNAs are often associated with heterochromatin regions and localized in certain chromosome positions (centromeric, terminal, and/or intercalary regions), allowing them to be used as probes in FISH procedures for comparative karyotype studies [47,74]. FISH-based patterns of the chromosomal distribution of satDNA clusters, in combination with 45S rDNA and 5S rDNA loci, facilitate the identification of homologous chromosome pairs in plant karyotypes, which is important for analyzing species diversity [47,74,75,76].

In our study, AmC9 presented unique clustered chromosome localization in the karyotypes of *Amaranthus*. The short repeat Am70 was localized in the centromere and terminal chromosome regions, which is typical for satDNA repeats [65]. FISH chromosome mapping of a combination of 45S rDNA, 5S rDNA, AmC9, and Am70, allowed us to identify chromosome pairs in the *Amaranthus* karyotypes and reveal species-specific patterns of chromosome distribution of these molecular markers. According to patterns of chromosome distribution of these molecular markers, 10 chromosome pairs were rather similar in the karyotypes of the studied species, confirming their common origin [34,35,36]. In the other five chromosome pairs, interspecies variations in chromosome localization and in the total number of AmC9 clusters were revealed. The minimal number of AmC9 clusters (6) was observed in the karyotype of *A. tricolor* (compared to *A. paniculatus* (7), *A. hypochondriacus* (8), and *A. cruentus* (10)), which could be related to the divergence of these species. The observed chromosome distribution patterns of these tandem DNAs showed that the genomes of *A. cruentus*, *A. paniculatus*, and *A. hypochondriacus* are more similar compared to *A. tricolor.* These findings are also in accordance with the previous taxonomic and phylogenetic data [22,35]. Moreover, the analysis of the karyotypes of five *Amaranthus* varieties indicated their genetic stability, since significant chromosome rearrangements were not revealed in their karyotypes. Our results demonstrate that the common satDNAs AmC9 and AmC70 together with 45S rDNA and 5S rDNA could be used as promising chromosomal markers for comparative karyotype studies within the genus *Amaranthus*.

Thus, the repeatome analysis and chromosome distribution patterns of the common DNA repeats (45S and 5S rDNA, AmC9, and AmC70) performed in *A. cruentus* var. Dyuimovochka, *A. cruentus* (accession number 842-04), *A. hypochondriacus* var. Kizlyarets, *A hypochondriacus* var. Krepysh, *A. paniculatus* var. Fakel, and *A. tricolor* var. Valentina demonstrate the species-specific nature of these patterns as well as the evolutionary instability of the repeat regions. This approach promotes further elucidating the mechanisms of the genomic evolution and differences in chromosomal organization of *Amaranthus* species.

## 4. Materials and Methods

### 4.1. Plant Material

**Figure 10:**
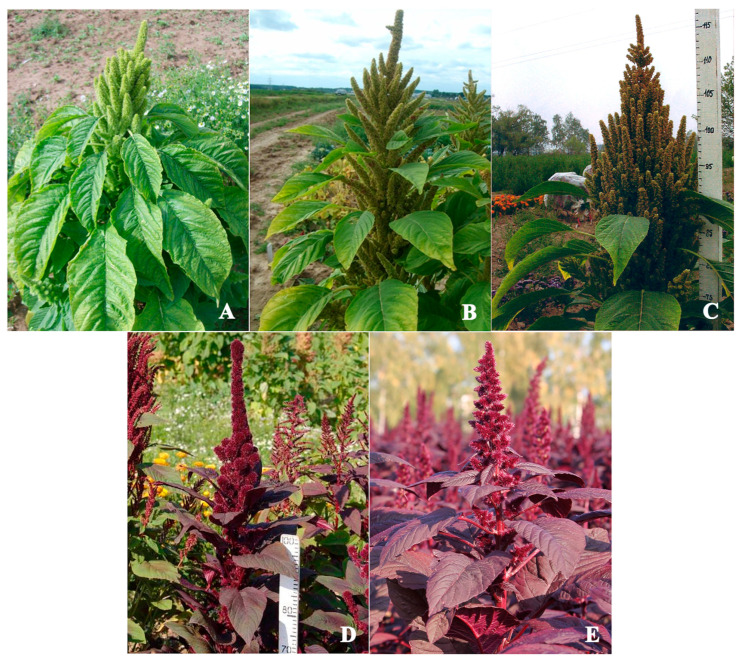
The plants of the studied *Amaranthus* varieties growing on the trial plots of the Federal Scientific Center for Vegetable Growing, Russia (**A**) *Amaranthus cruentus* var. Dyuimovochka; (**B**) *Amaranthus hypocondriacus* var. Kizlyarets; (**C**) *Amaranthus hypochondriacus* var. Krepysh; (**D**) *Amaranthus paniculatus* var. Fakel; (**E**) *Amaranthus tricolor* var. Valentina. The images were taken by M.S. Gins.

Seeds of one *A. cruentus* specimen were obtained from the seed collection (accession number 842-04) of the All-Russian Institute of Medicinal and Aromatic Plants, Moscow, Russia. Seeds of five *Amaranthus* varieties (*A. cruentus* var. Dyuimovochka, *A. hypochondriacus* var. Kizlyarets, *A hypochondriacus* var. Krepysh, *A. paniculatus* var. Fakel, *A. tricolor* var. Valentina) were provided by the Federal Scientific Center for Vegetable Growing (Moscow region, Russia), where they were developed and cultivated (Figure 10, Table 1).

### 4.2. Bioinformatic Sequence Analysis

The bioinformatic analysis of the repeatomes of *A. tricolor*, *A. cruentus*, and *A. hypocondriacus* was performed using RepeatExplorer2/TAREAN/DANTE_LTR pipelines based on the Galaxy platform (https://repeatexplorer-elixir.cerit-sc.cz/galaxy/, accessed on 26 April 2025) [77,78,79,80].

For genome-wide comparative analyses, the publicly available sequencing (Illumina platform) data of *A. tricolor* (accession number SRR21968682; https://www.ncbi.nlm.nih.gov/sra/SRR21968682, accessed on 18 April 2021), *A. cruentus* (accession number SRX10357816; https://www.ncbi.nlm.nih.gov/sra/SRX10357816, accessed on 18 April 2021), and *A. hypocondriacus*, (accession number ERR3021343; https://www.ncbi.nlm.nih.gov/sra/ERR3021343, accessed on 14 January 2020) were used.

From basecalled sequencing data, RepeatExplorer2/TAREAN selected and filtered by quality 85,873,434 paired-end reads (150 bp in length) (*A. tricolor*), 25,000,000 paired-end reads (150 bp in length) (*A. cruentus*), and 36,000,000 paired-end reads (100 bp in length) (*A. hypocondriacus*). Then, 452,669 (*A. tricolor*), 1,321,949 (*A. cruentus*), and 2,503,965 (*A. hypocondriacus*) high-quality reads were randomly selected for further analyses. This corresponded to the coverage of genomes of *A. tricolor* (1C = 520 Mb), *A. cruentus* (1C = 370.9 Mb), and *A. hypocondriacus* (1C = 466 Mb) [34,35,36,54], which was recommended by the developers of these pipelines (0.01–0.50×) [78].

RepeatExplorer2/TAREAN was launched with the preset settings based on the Galaxy platform (https://repeatexplorer-elixir.cerit-sc.cz/galaxy/, accessed on 26 April 2025). Each repeat proportion was calculated as a ratio of the number of reads specific to the particular repeat type to the sum of all reads used in the cluster analysis. The sequence homology of the identified tandem DNA repeats was estimated using BLAST (version BLAST+ 2.16.0) (NCBI, Bethesda, MD, USA). According to BLAST results, the heatmap was created using the graphic heatmap function of the Seaborn data visualization 0.13 2 Python library.

Based on two satDNA repeats identified in the *A. cruentus* genome (high-confidence AmC9 and low-confidence AmC70), oligonucleotide FISH probes were generated using the Primer3-Plus software (https://www.primer3plus.com, accessed on 2 April 2025) (Table 2) [81].

### 4.3. Chromosome Spread Preparation

Amaranth seeds were germinated for 3–4 days at room temperature (RT) on moist filter paper in Petri dishes. Root tips (0.5–1 cm long) were cut off and treated with a saturated aqueous solution of α-monobromonaphthalene (20 mL of α-monobromonaphthalene was mixed with 3× volume of distilled water) at RT for 4 h to accumulate mitotic cells with increased chromosome sizes (Supplementary Figure S1). The root tips were fixed in the ethanol/glacial acetic acid (3:1) fixative at 4 °C for 48 h and then transferred into the 1% acetocarmine solution in 45% acetic acid for 15–20 min. After that, each root was placed on a glass slide and squashed using a cover slip. After freezing in liquid nitrogen, the slides were dehydrated in 96% ethanol and air dried.

### 4.4. FISH

For sequential FISH assays, a combination of four labeled DNA probes was used. Two wheat DNA probes, pTa71 containing 18S-5.8S-26S (45S) rDNA of common wheat [82] and pTa794 containing 5S rDNA of common wheat [83], were labeled directly with Aqua 431 dUTP or Red 580 dUTP fluorochromes (ENZO Life Sciences, Farmingdale, NY, USA) using the Nick Translation DNA Labeling System 2.0 (Life Sciences Inc., Syracuse, NY, USA). Two oligonucleotide DNA probes, AmC9 and AmC70, were synthesized and labeled with nucleotides ROX-dUTP or 6-FAM-dUTP in *Syntol* (Moscow, Russia).

FISH assays were carried out according to the previously described protocol [47]. Briefly, chromosome slides were pretreated with 1 mg/mL of RNase (Roche Diagnostics, Mannheim, Germany) in 2 × SSC at 37 °C for 1 h. Then the slides were washed three times in 2 × SSC for 10 min each at RT, dehydrated in the graded ethanol series, and air dried. Next, 40 ng of each labeled probe was dissolved in the hybridization mixture (containing 50% formamide, a total volume 15 μL) and dropped on each slide. The slides were sealed with rubber cement under coverslips, co-denatured at 74 °C for 4 min, cooled on ice, and hybridized overnight in a moisture chamber at 37 °C. After that, the slides were washed in 0.1 × SSC and then in 2 × SSC (for 5 min at 42 °C each) followed by a 5 min wash in PBS at RT, dehydrated in the graded ethanol series, air dried and stained with 0.1 μg/mL DAPI (4′,6-diamidino-2-phenylindole) (Serva, Heidelberg, Germany) in Vectashield mounting medium (Vector laboratories, Peterborough, UK).

### 4.5. Analysis of Chromosome Slides

For chromosome analysis, an epifluorescence Olympus BX61 microscope with a standard narrow band-pass filter set and UPlanSApo 100/1.40 oil UIS2 objective (Olympus, Tokyo, Japan) was used. Chromosome images were acquired with a monochrome charge-coupled camera (Cool Snap, Roper Scientific, Inc., Sarasota, FL, USA) in grayscale channels. The obtained images were pseudo-colored and processed using Adobe Photoshop 10.0 (Adobe, San Jose, CA, USA) and VideoTesT-FISH 2.1 (IstaVideoTesT, St. Petersburg, Russia) software.

Two specimens of each species *A. cruentus* and *A. hypochondriacus*, as well as one specimen of each species *A. tricolor* and *A. paniculatus*, were used for the cytogenetic analysis. At least five plants and fifteen metaphase plates from each sample were examined. Chromosome pairs in the karyotypes were identified according to the previously reported classification [47], which was based on the localization of the chromosome markers and took into account the chromosome size and morphology.

## Figures and Tables

**Table 1:** Characteristics of the studied *Amaranthus* varieties.

Variety	Leaf Color	Inflorescence Color	Stem Color	Vein Color	Seed Color	Usage
*A. tricolor* var. Valentina	purple	purple	purple	purple	black	leafy vegetables, grain crops
*A. cruentus* var. Dyuimovochka	green	green	green	green	black	leafy vegetables
*A. paniculatus* var. Fakel	green with red	purple	green with pink	pink	black	fodder and ornamental plants
*A. hypochondriacus* var. Kizlyarets	green	green with red	green	green	white	grain crops, fodder plants
*A. hypochondriacus* var. Krepysh	green	green with red	green	green	white	grain crops, fodder plants

**Table 2:** The generated oligonucleotide FISH probes.

Tandem Repeat/Genome Proportion [%]	Sequences of the Generated Oligonucleotide FISH Probes
CL9/0.55	AmC9 CATTGTTCATTGATCATTGATCCTTGTTCATTGTTCATCGTT
CL70/0.1	AmC70 AGGGTTTAGGGTTTAGGGTTT

## Data Availability

All data generated or analyzed during this study are contained within the article and Supplementary Materials.

## Supplementary Materials

The supporting information can be downloaded at: https://www.mdpi.com/article/10.3390/ijms262211026/s1.
