# Supplementary material for: Comparative Analysis of Chromosome Repeat DNA Patterns in Four Amaranthus Species
Source: Int J Mol Sci. 2025 Nov 14;26(22):11026. doi: 10.3390/ijms262211026 (PMC12651990; doi:10.3390/ijms262211026)

Supplementary materials

Supplementary Table S1. Proportions of major DNA repeats identified in genomes of *Amaranthus tricolor, A. cruentus* and *A. hypochondriacus*.

**Repeat Name**	**Genome Proportion, %**		
	A. tricolor	***A. cruentus*[1]**	***A. hypochondriacus*[1]**
Retrotransposons (Class I)	14.41	11.06	10.07
Ty1 Copia	7.19	4.28	4.00
Ale	0.30	0.29	0.29
Angela	0.08	0.12	0.13
Bianca	0.27	0.12	0.11
Ikeros	-	0.05	-
Ivana	0.15	0.06	0.06
SIRE	5.55	1.43	1.62
TAR	0.11	0.86	0.86
Tork	0.73	1.35	0.93
Ty3 Gypsy	5,83	5.37	3.06
non-chromovirus Athila	0.15	1.44	1.07
non-chromovirus Tat-Retand	0.80	0.53	0.41
chromovirus CRM	0.38	0.49	0.28
chromovirus Tekay	4.50	2.90	1,30
chromovirus Reina	-	0.01	-
LINE	0.41	0.54	0.66
Unclassified ME Class I	0.98	0.87	2.35
DNA Transposons (Class II)	5.04	3.52	3.07
CACTA	1.73	0.82	1.24
MuDR_Mutator	1.99	1.46	0.77
hAT	0.96	0.53	0.39
PIF_Harbinger	0.07	0.06	0.05
Tc1_Mariner	0.29	0.42	0.38
Helitron	-	0.23	0.24
Ribosomal DNA	1.72	5.06	3.54
Unclassified repeats	13.57	11.5	15.21
Satellite DNA	5.70	0.27	0.16
Putative satDNA families	4 high confident<br>2 low confident	7 high confident<br>4 low confident	2 high confident<br>6 low confident

[1] These data were taken from our previous study [47].

Supplementary Table S2. High confident putative satellite DNA repeats identified in the genome of *Amaranthus tricolor* using RepeatExplorer2/TAREAN.

**Tandem Repeat/ Genome Proportion, %**	**Repeat Length, bp**	**BLAST Similarity**
AmT1/12.0	169	100%/81% of coverage/identity with AmT129 in *A. tricolor*<br>96%/91% of coverage/identity with AmC4 in *A. cruentus*<br>100%/89% of coverage/identity with AmH9 in *A. hypochondriacus*<br>100%/84% of coverage/identity with AmH51 in *A. hypochondriacus*
AmT2/9.7	42	86%/92% of coverage/identity with AmC27 in *A. cruentus*

		67%/96% of coverage/identity with AmC9 in *A. cruentus*<br><br>60%/96% of coverage/identity with AmH51 in *A. hypochondriacus*
AmT18/0.4	178	No results
AmT115/0.034	685	No results

Table S3. Low confident putative satellite DNA repeats identified in the genome of *Amaranthus tricolor* using RepeatExplorer2/TAREAN.

**Tandem Repeat/ Genome Proportion, %**	**Repeat Length, bp**	**BLAST Similarity**
AmT129/0.029	683	95%/81% of coverage/identity with AmT1 in *A. cruentus*<br><br>85%/75% of coverage/identity with AmC4 in *A. cruentus*<br><br>92%/78% of coverage/identity with AmH9 in *A. hypochondriacus*<br><br>49%/72% of coverage/identity with AmH51 in *A. hypochondriacus*
AmT193/0.015	41	No results

Supplementary Table S4. Putative LTR repeats identified in the genome of *Amaranthus tricolor* using RepeatExplorer2/TAREAN/ DANTE_LTR.

**Tandem Repeat/ Genome Proportion, %**	**Repeat Length, bp**	**DANTE_LTR Results**	**BLAST Similarity**
AmT14/0.51	7379	RT Class_I	No results

T Supplementary able S5. Sequences of the DNA repeats identified in the genome of *Amaranthus tricolor* using RepeatExplorer2.

**Tandem Repeat/ Genome Proportion [%]**	**Repeat Length, bp**	**Sequence**
AmT1/12.0	169	TGGCACTTTTGCGCATAAAGTAGCTCAAACTTGGTTTGTTTTGCACGAAACTTGGC<br>ACACAACACTATTTGGTATATATTATTGTGTTGAAGTGGTTAGAATTGAAAATCAT<br>AGTCATATGCTAGAAATTACGTGTTAAGTTGCGATTTTAAGGGTTTTTAAGCGTTT<br>T
AmT2/9.7	42	CATTGATCCTTGTTCATTCTTCATCGTTCATTCGTCATTGAT
AmT18/0.4	178	AAAAACTAATCAAAAATAATAAACAAAATCAAAATCAGATCCAAACAGAACCA<br>TTTACATGAGTATGACACAACATAAAACTACAACAAAACAGATCAAAATGAATA<br>AACAAAATAAACAAATTGCAAGCCTCAAAAGGATAGGAAAAATCAGATCAATA<br>TTAATAAAATAAAAAATC
AmT115/0.034	685	TACATAAAGTTAATACATAAGGGTGTATATAAAAGAAATCAAAAAAATATTTTCT<br>ACCATAACATAATTATTAATTTTTCAAATAGAGTTATTGAATAATTAGTGTTATTA<br>ATATTTCTACAATTGTTTCTACATAATCAAATGAAATATATACTATAAGTAACGGT<br>TATTTTAAAAAAAATTATATAAACAATTTAATTAATATTTAATTTACTGATTTAAA<br>TAGTTTTTCAACTGCACGCACGATGTGATTTAAAAAATTTATAACATATAGTGCTT

		TTATTTTTAAAATTATAACTTTTAAGAAAAATTTAGGTGATTTTCCAACAATGTTAT<br>CATAAATAATTTTAGTCATTCCCCAAATAATTTTTGACTATCATGTATAAATTTAC<br>ATAATTATTAACAAAATTTTTTGATTTGCGAAAATCCGAGAGATGAAAAAAAAGA<br>ATTCATTTTATTTTGGTATTTACTTTTTTTAGGCTTAATGTTAGAGAAAATAATTTTT<br>TAAGTTCTTCTATAAAATATGAAAAAGTGCAAATGTAAGTCACATTGTTTTATTAT<br>ACTGGATAATGTAAATTGCAAAATAAACATAAATGGAAAAAGATTGAGTGAATT<br>GAATATTTATCTAAAAGAATCAAGTGAAATACAATACAACTTTATAAATATAATA<br>AAAAAATAATTTTTC
AmT129/0.029	683	CTTAAAAATCTGTCAAATGCAACTTAACACGTAGTTTTAAGAATATTAGCATTGTT<br>TTATATTTTAACCATATCAACACAATATTATATACCAAATAGTGTTGTGTGCCATG<br>TTTCTTACCAAACCAACCATGTTTGAACTCCTTTATGCGATAAAGTGCAAAAACA<br>CTTAAAAAAACATAAAAACATAAAAACAAAACTTAACACGTAATTTCAAGCATT<br>TGACTATTGTTTTTAATTATAACCAATTCAACACAATAATATATACTAAATAGTTT<br>TATGTACCATATTTCATGCAAAACAAACCAAATTAGAAGTATTTAATGCTTAAAA<br>GAGCTAGAAACGCATAAAAAAACCATTAAAACGCCACACAGCACGTATTTTCAA<br>GCATATGACTATTGTTTTTAATTCAACACTTTTCAAAACAATGATACATACCAAAT<br>AGTGTAGTCTGCCTATTTGAATTGCAAACAAACGAAATTTGTACCAATTTATGCG<br>AGAAATTACAAAAAACGCTTAAAAAACATTTAAAACGCAACTTAGCATGTAATT<br>TCAAGCATATGACTTATGCCTTCAGATTTAACCATTTCCACACAAAAATATATACC<br>AAATAGTGTTGTCTGTAAGATTGAATGCCACACAAACGAAGTTTGCACGACTTTA<br>TGCGACAAAGTGCCAACAACG
AmT193/0.015	41	GAGCTTAGAAGGAAAGAGTTAGGCTTATAGATAGTAGTTTA
AmT14/0.51	7379	GTATCAGAGCCAGTAAGTTATTAAATTGATTAATTGTGCATTTATTAAATTGCATT<br>TGTATTGAGTGTTGGTAAAGTGCTTGGTCAGCCTAATTAAGTTGTTGGTAAGCCAT<br>TTTAAGTCTTGGTGTTCTCGGTTAGGTGGTCCCACTGTTCCCATTCAATTCAGTCAT<br>ATTTAGTTTATGCAACATTTAATTTATTAATGGCAAACTGGTTAAGGAAAGCTATT<br>AGTTTAGGATCACCTAATAATATAATTCAAAGTAGTGAAGTTCATATAGAAAAGA<br>ACTTTTCAAATTGGGAAATTCCAAAAGAAAATCTTACAAAAATTTATCATATAGG<br>CATTACTGATTTTAAGACTGCATTTAGCATCAAAACTCATGAAGAGACTAGAAGC<br>ATTCAAAAAGAATCTGAAACTATGCATTTACTTTCAGAATCCAGCATTAAAGCTT<br>ATCTCAAACACAGATTTAGATTCATTCATTTTGGCCTTGTTCAAATAGGAATAAAA<br>CCATTAGTTCATAAAGGAGTTGATTGTCCAATTTTCCTAGCTCTCAGAGACGATCG<br>ACTTAATAATTATAAAGATTCTGTTTTGGCTATGATTCAAACTAATATCTGTAATG<br>GCCCAATATATTTCAATTGTTTTCCAAACTTTACAGTAGATCTTCAAGACCCATTA<br>ATCTTATCTTCATTAATTTTAGATATTCAAATTTTAAATAATAAATTTAAAGAGTTT<br>GCTAGAAATTTTGCAATTGTTTTCCGTGTTTATTTTAGATTAATGAATTCCACAGTT<br>AATCCAAAATTCATACATGAGGCTTCTTCAAAAGAAGAAACTATTTTTATAGAGG<br>TTCATGCTGAGAATACTAAATCAATAGTTTCTACAGCAAAAACCTTGAGATGGGA<br>TCAAATTTCTATTCCTGAGACATTTCAATTCTCAAATCCAATGCCTCCTAGAAACA<br>TAAGACAAGAAATAGAATGCATTGAGGAAGACGAAAATAAAGTTCTTATAAGGT<br>TTGGGTCTATGAGAGAATTATCTAGTTCTCCTTGTACTTCTTCTTCCAACTATCCAC<br>GAAGATCTTTTTCTTTTCCTCAAAAAACAACTTCAGTCATTTCTGAACCAATCAAA<br>GTCAGATATAAATCTCCTATTCCTGAATATGAAGCAAATGCTTCTCAAACTTCTCA<br>AATCTCTTCATCTAGACCTGCATCTCCCACAATGTCTGATATGCAAAATTTTGACT<br>CTATCAATACAATTACTAAAGATTTTGAAATAGATAAAAACTATCTAAGAGATGA<br>ATTTAATTCCTCTCACAATGCTGAACATCGAGATTGGTTTAGATCTTTTTCTCGCTC<br>CTTTAAAGACTCAATAAGAGATGTATGGTATAAAGATATGAATACTATTCAAATT<br>AATATCCCGTTTTTTACATGGTTTTTATTGTTTTGTGAAAAACAAGGTATTGCTAAT<br>CCATATTCTCAAAATCACATATTTGTACAAACCAAACTTCAAAAAAATTGGGTTT<br>TGAAGGATGGACAAACAATTAGTTCAGTTCATCCACCATTACAAAATATTACTTT<br>TCCCTATTATGACCGTGAAATAAAGGCTAGTCCTTTTAAAGAAGGAAGAGTAGAT<br>CAAACAGATAAAACTCCAAATGTAGAAGACATCATTAAAATTTATCATCAAAAT<br>AATTTTACAAATCAAATCCTTCATACCGTTGCTACACAAGTAGATCAACTTTCAA<br>ATAAGGTTGATAATTTACAAAAAGAAACCTCTAGATCCATAGCCCCTAATACGAT<br>TGCATCATTAGGATCTAATAAGGTTTCTGCACCGCATTTCAAACCACCAGGATTA<br>ACTATACCTATGACTCAAATGTTTCCAAAAAATGGTTTTACTTCTTCTAACTCTAA<br>TTCTGCAGTGTTAGATAAAATTCAAAAATCTTTACAAAAATTAGCAGATAGTCCA

		AGTCATATTAAGGTTTTGAGTAAATATCAAAGTTCAGAGATATCTTCTGATGAAG<br>AAAATTCTGAGTCTTTAATCATTTCAAAAGTTGCAAAGCAATTTCAAGAATCTGA<br>TAATGATCCTCATCAAATTAATAAAATACGATCATCTTGGAAGAATATCCCTACA<br>AAAAACTATTATCCTAGGCCTACTCCTGTTGATCTTCAATATGAAGAAAGGTCTA<br>CATTTACCTCCAAATCCTTTTCCCCAGATATGATCCATGAATGGAACATAGATGG<br>AAAATCTGATTATGAAATTCTTAATACTCTTCAAAATATGGGAATGGCTATTGTAG<br>CTTACAAGGCAAAACCTTTGGATGAACAAATAATTTTTGGTTTTATTATATCAGGT<br>TTTACTGGGCAATTAAAAAACTGGTGGGATAATCTTCTCACTTTACAAGACAGAT<br>TAGAAATTTTGAATCACGTTACTGACATCATGGATGATCAAGGAAATGTATTTCA<br>AAAATCTGACTGCTGTGATTTCTTAATCATAGTTATTGCCTTTCATTTTGTCGGTAA<br>CCCTACTCAAACTCTTTCTTCTGGAGAAACGATTTTACAAAATTTACGTTGCCCAA<br>CATTAAGTGACTATAGATGGTATAAAGATACCTTCTTTAGTTATGTTTTACAAAGA<br>CAAGATTGCAATCAAAGTTTTTGGAAAGAAAAATTTATATCAGGACTACCAAAAT<br>TATTTGCTCAAAGAATTTTTAGAAAAATAGCTGAACATGATACAGATAAAGAAC<br>AAGCTCTTAATAATACTACTTATGGAGGACTATTCGCTTTCATTAAAAAGGAAGG<br>TCTTTCTTTATGTGAAGAACTCAAACTCCAAGCCAAATATACCTCTGAAAAGAAA<br>CAATCTAGGAAAGAAATTGGATGTTTCTGTGAAGCATTTGGTATGGAAGCTATTA<br>GAGCTCCCTCTACAAAAAAGAAAATTAAAAAACAATTTTCTAAATTTTCAAAAG<br>ATAAAAATAAAAATTCTTATCCTTCTTATTATCGAAAGAAACGAGGAAAGAAAA<br>ATTTCGGAAATTTCCGAAAATCAAACAAAGCTTTCAAAATCTTTTGTTATTCGTGT<br>GGAAAACCTGGACATAAAGCAAATGCTTGTACCACAAAGAAAAAGATTCAAGA<br>ATTATTTATTAACGACTCTGAGCTAGGAGATAAAATATCCAAAATATTATTACAA<br>ACCCCTGAGTCTACTTCTTCTTCTTCTTCAGAAAAGGAAGATGACGAAATTTTACA<br>AATTAATGATCAAACTTCTGATTCTGAATCTTCGATTTCTTCCTCTCCTATCAAATG<br>CATTAATGTTTTAACAGATAAAGAGAAAAATGTGGAATTTCTTTTTGATCTCGTTG<br>ACAAAATCCAAGATAAAGAGATCAAAAGAGAAACCCTTTTGAAACTCAAAACTA<br>TGGTTTTGGGTGAAACCTCTACCTCTAAAAACAAAGAAAGTATTCCAATTCCTCA<br>AATTGAACCTTTCAATATTTCAAAATTATTAGAAAAATACTCTTCCAAAGAAATG<br>ACTTCTAAATTAATTCCTATTAAGAATCAAACTCTTTCGGTCAAAGATCTTCAACT<br>TGAAATAAATAAAATCAAAGAAGACATCATTTCTATCAAAAATAGTTTAAAGCA<br>TTTAGAAATCAAAGATTTCGAACTTGAAACAAAATTATCTATTTTAGAAAACCCC<br>ATCTATAAAGAAAATCTTTCTTTGGGAAAAGAGAAAGTTAATGGGGATGATGATG<br>ATTTTATCACTGTTATTAATAAAATTAATTTTCAAAAATGGTATGCACCAGTTACT<br>TTTCAAATTGGAGATTTTCAAAAAACCTATATTGCTTTAATTGATAGTGGAGCGGA<br>TCAAAATTGCTTAAGGGAAGGATTAGTCCCAACCAAATTTTATGAAAAAACAAA<br>AACTCGGTTATATAGTGCAAATAGTTCACCCATGGATATTAAATATAAAATTTCA<br>AAAGGAAAGATCATAAATGAAAATTATAGTTTTACTAATACTTTCATTATTATTAA<br>TGATATTAAGGAAGAAATAATATTAGGCACTCCCTTTTTAACACAAATTTATCCTT<br>TTTGGGTTGATGACAAAGGTGTTCATACAAAAATTTTAGGGAAACAATTATCATT<br>TCCTTTTTTAAGTCCCATGTTACAAAAAGATATTAATTTATTACAAAATTCTTCAGT<br>AATACAAAGTATAAATTTAATAAAACAAAAAGAATTTCAAATTTCTTCTTTAAAA<br>GAAGAAATTTCTTTTCAAAGGATTGAAGAACAATTACAACAATCAAAATTGCAA<br>CAAAAAATTTTTAATTTAGAAAATATTTTTAAATCAAAAGTTTGTGCTGATATTCC<br>AAATGCCTTTTGGGATAGAAAAAGGCATATTGTCACATTGCCATATGAAAAAGAT<br>TTCTCTGAAAAAAATATTCCTACAAAAGCTAGACCCATCCAAATGAATTCTGAGC<br>ATCTTGAATTCTGCAAGAAAGAAATTCAAACTCTTTTAGATAAAAAATTAATAAC<br>TCCATCCAAATCTCCTTGGAGCTGTGCAGCATTTTATGTTATGAATGCAGCAGAA<br>AAAGAAAGAGGAGTTCCTAGATTAGTTATTAATTACAAACCTTTAAACAAAGTTC<br>TTCAATGGATAAGATATCCAATCCCAAATAAAAGAGATTTAATTAATCGCTTATA<br>TAAAGCCAAAATATTTTCAAAATTTGATATGAAATCTGGTTTCTGGCAAATACAA<br>ATTGCTAAAGAAGATAGGTATAAAACTGCATTCACGGTACCATTTGGACATTATG<br>AGTGGAATGTAATGCCTTTTGGACTTAAAAATGCTCCTTCTGAATTTCAACATATA<br>ATGAATGATATTTTCAATCAATTTTCTTCATTCATAATTGTTTATATAGATGATGTT<br>TTAATTTTTTCTCATGATATTGATCAACATTTCAAACATTTACATATATTTCTAAAC<br>ACTGTTGAAAAAAATGGTTTAGTAGTTTCTGCCACTAAAATGAAATTATTTCAAA<br>CAAAAATTAGATTTTTGGGACATGACATTTATAAAGGAACAATCAAGCCAATTAC<br>AAGGTCTTTAGAATTTGCAGAAAAATTCCCTAACGAAATTAAAGACAAAACCCA<br>GTTACAAAGATTTTTGGGATGTCTAAATTATATATCAGATTTTTTCCCAAAGCTCA<br>GACAAATTTGCATGCCCCTCTTTCAAAGACTTCAAAAAAATCCGCCTCCCTGGTC

		AGAAATTCATACTAAGACTATTATTGAATTAAAACAAAAAATCAAATCACTTCCA<br>TGTCTAGGCATTCCAGATCCATCTGCATTCATGATAGTAGAAACAGATGCATCAG<br>AAATAGGATATGGTGGCATATTAAAACAAAGAATAGATACAAAAGAACAATTA<br>GTTAGATTTCATTCAGGTCTCTGGCTCGGACCTCAAAAAAATTATTCTACCATTAA<br>AAAGGAAATTTTATCTATAGTACTATGCGTTTCAAAATTTCAAGATGATCTTTACA<br>ACAAAAAAATTTTTGATAAGGATCGACTGCAAATCTGCAAAAGAGGTTCTAGAAA<br>AGGATGTCAAAAACATTGTTTCTAAACAAATTTTTGCAAGATGGCAAGCAATTTT<br>ATCAGTATTTGATTTTCAAATAGAGCATATAAGAGGCGAAAGTAACTCTCTCCCT<br>GATTTCTTAACCAGAGAATTTCTACAGGGACATCATAATGCCAAGGAAGAAGAA<br>TCAGCAAGAAAAACTCAAAGGGTTCCAGAGTTCAGATGATTACGAAATGGAACC<br>AGATCCAAAACCAAACAAAACAGAACAAAATAAATTTTCATCCTCCAAAAATCT<br>TCAAACTACCCTATCCATTTCAAACAAATTTACTCAACTAAATGATTTTCCCCCAC<br>TTCCATATTCACAAATTGTTCAAGAGCCTACTTCACAAAATATTATTTCAAATTCA<br>AAACAAAAAGAACCAACTACAAAAATACCAACACAAAGCTTTACCAAATCTGC<br>ATACATTACAAAAACCGAAATAGAAAATGTATATCTCACTAATTTTACAATTCCT<br>CAAAGCCCACAAATTATTAATTCCATCAATCAAAAAACTTTTCCAAAAGGTTGCG<br>AATGGATACCAGAAAATCGTTTTAAAACTCAAAGGTTTTATGAATTTATTTTAGTA<br>GATTCAAACTCTGTTGAAATCCAACATATCAAGGATTCTTCAGAAAAAATAATTT<br>ATTCAAAATGTATTTTTAAGAAAATTATTTCGTCTGTAGAATGGGGAGACCCTTAT<br>GCAGAACGAATTTTTTCAAAAAGATTTTTCCCTCAAAATTACTCATACTATGATTA<br>CAAAATGGCCTGGTATAGAGCATTTTTATTCAGACCATTTGATCATTCATGGTTTT<br>TTATATTTGACAAATATTGTCCAAAGAAATATCCTATGTGGTTTTATCATTGGTGG<br>TATTTATTTGGACCAACTCCTCAAATTTATCCAAAAGAATGCAATGAGGGTTTCAC<br>AACTTTTGTTGCCAATTCAAATTATCAAGCTTATGAAACTCCAGTCATGTTTCATG<br>CAGAATTTAAAATTCCATGGATTCTATGTTGGAGTTACTGTCTCAAACAACACCTT<br>CCACGTCCATATCCATATTCATTAATAAGAGAATTCAAAATTAAATGGTGGGACA<br>AATTTACCTTAGAAATTTGTTCAACAAAAAAATGTTATCTCCTTTTTTCAAACCGGT<br>ATAAAAATGGAATACCAATCTCCATCAACCACCAACAAACCTTCACCCTCAAAA<br>GCAACATCAAATTCAAACTCTCCTCAACACAATAAAAATCCCAAAGTAGAAAGT<br>CCAAATTCAGAATCATTATCTAAAGTCCAGAAAGCTATTCTCAAAAAGATTCTTG<br>AAGATCCACAATATGCAAAGAAAATATTATCAGAAGAATCCTCTGATGAGATTTT<br>ATCGGAGTTTTCAAATGAGTCTTATGATCCAACATTTGGTGGCCCATTTGCTCAAG<br>ATCCATACGACTTTTAATATTGAGTTCACAAAAGTTCAATTCAAATGTTCAACAA<br>CTCAAATTAATTCATCCGGAAGCACAAACGTAAGCAATGACGAAATCAAAAGCA<br>TCTCCAACAGTAATTCAAAGCATCTTCTACAGTATTATAGTGTAACTTTAATAATT<br>ATGTTTGTACTATTTCTCTTTAATTTCTTATTGGAAGAGCAGATTGCACCTTATCTC<br>TTCCTTTGTGTATAAATAAAGTGTCTAGCTCTAAGATGAGAGCATCCTGTAATTTT<br>CTTTTGTTTAAGTCTTATCTCAAATAAATCTAACTTTCTTTACTTATCTTATTATTAT<br>CAATTATATAATAAGATTTATATCCACAAAATTATTAAATTATTTTCTCTATCGAT<br>CCTGTACTCCCTATTG

Supplementary Table S6. High confident putative satellite DNA repeats identified in the genome of *Amaranthus cruentus* using RepeatExplorer2/TAREAN.

Tandem Repeat/ Genome Proportion, %	Repeat Length, bp	BLAST Similarity
AmC4/1.0	169	100%/98% of coverage/identity with AmH9 in *A. hypochondriacus*<br>99%/85% of coverage/identity with AmH51 in *A. hypochondriacus*<br>96%/91% of coverage/identity with AmT1 in *A. tricolor*<br>100%/77% of coverage/identity with AmT129 in *A. tricolor*
AmC9/0.55	42	100%/93% of coverage/identity with AmC27 in *A. cruentus*<br>93%/100% of coverage/identity with AmH4 in *A. hypochondriacus*<br>67%/96% of coverage/identity with AmT2 in *A. tricolor*

AmC27/0.23	42	100%/93% of coverage/identity with AmC9 in *A. cruentus*<br>100%/91% of coverage/identity with AmH4 in *A. hypochondriacus*<br>86%/92% of coverage/identity with AmT2 in *A. tricolor*
AmC123/0.047	154	No results
AmC217/0.014	470	21%/74% of coverage/identity with AmH3 in *A. hypochondriacus*<br>*Vitis vinifera*, whole genome shotgun sequence, contig VV78X037039.33, clone ENTAV 115<br>*Malus domestica* cultivar Golden Delicious chromosomes 1, 5, 7, 9, 14, 16, 17
AmC239/0.012	683	No results
AmC246/0.011	271	26%/84% of coverage/identity with AmH209 in *A. hypochondriacus*

Supplementary Table S7. Low confident putative satellite DNA repeats identified in the genome of *Amaranthus cruentus* by RepeatExplorer2/ TAREAN.

Tandem Repeat/ Genome Proportion, %	Repeat Length, bp	BLAST Similarity
AmC32/0.2	4950	83%/97% of coverage/identity with AmH27 in *A. hypochondriacus*<br>13%/73% of coverage/identity with AmH6 in *A. hypochondriacus*<br>*Amaranthus palmeri* extra chromosomal<br>*Amaranthus palmeri* clone NJ_Ap_15, complete sequence<br>*Amaranthus palmeri* clone NJ_Ap_14, complete sequence
AmC70/0.1	21	No results
AmC103/0.062	88	No results
AmC154/0.027	154	No results

Supplementary Table S8. Putative LTR repeats identified in the genome of *Amaranthus cruentus* by RepeatExplorer2/ TAREAN/ DANTE_LTR.

Tandem Repeat/ Genome Proportion, %	Repeat Length, bp	DANTE_LTR Results	BLAST Similarity
AmC5/0.82	4924	No results	*Amaranthus palmeri* clone NJ_Ap_6, complete sequence<br>*Amaranthus palmeri* extrachromosomal
AmC12/0.47	3008	Class_I|LTR|Ty3 Gypsy|chromovirus|CRM	41%/93% of coverage/identity with AmH26 in *A. hypochondriacus*

Supplementary Table S9. Sequences of the DNA repeats identified in the genome of *Amaranthus cruentus* using RepeatExplorer2.

**Tandem Repeat/ Genome Proportion, %**	**Repeat Length, bp**	**Sequence**
AmC4/1.0	169	ACACTATTTGGTATATATTATTGTGTTGAAGTAGTTAGAATCGAAAATAATTGTC<br>ATATGCTTGAAATTAAGTGTTAAGTTGCGTTTTTAAGGGTTTTGAACTATTTTTGT<br>CACTTTCGCGCGTAAAATAGCTTAAACTTGGTTTGTTATGCACGAAACTTGGCA<br>CACA
AmC9/0.55	42	CATTGTTCATTGATCATTGATCCTTGTTCATTGTTCATCGTT
AmC27/0.23	42	TCAATGATCAATGAACAATGAACGATGAACAATGAACAAGGA
AmC123/0.047	154	TCTCAAATCACTCAAGAAAAAAAACCCATTACATTCAAAAAATTACCCGAACA<br>ACAAAAATAACATACAAAAATAGCATTTATGATTCTTGAACAACTTATTTAATT<br>AATGAGTATAACATCATGATTCATGAAACACTAATAATAGTTACCAC
AmC217/0.014	470	TGAGAAAGTTTGGGTATGTGCAAAGCAATTCAGATCATACACAGTTCTTAAGA<br>AAAATGATACGTGAGAAAAGTGTATAATTCCCGGGAGAAAGAAAGCTGTAGG<br>TTGCAAATGAGTGTTCACAATTAAATATCATGCTGATGGAACCATTGAAAGATA<br>TGAAGCTCGACTTGCAGCTAAAGGGTGCACTCAAACATATGGGGTAGATTACT<br>CTGAGACTTTTTCCCCGGTTGCCAAAATTGATACTATTAGAGTGTTGTTCTCAAT<br>AGCAGCAAACAAAAACTGGCCCTTATATCAGTTTGATGTGAAAAATGCCTTTCT<br>TCATGGGGAGATTGAAGAAGAAGTTTTTATGATTGCTTCTCTTGGATTTAAAAA<br>TGAGTTTGCATCAGGAGAAGGTTGTAAACTCAAGAAGGCTCTTTATGGCTTAAA<br>ACAATCCCTTCGAGCTTGGTTTAGAAGATTCACCTCAGCAA
AmC239/0.012	683	TTTTAAGTATTTTCTTGTTTTTAAATGAACCACATGGGTTTCACTTGCCTTCATTA<br>GGAGTTTAGAGTTTGTATTTCTTTATTAGTCGTTATGTTAGAGTGTGTGTTTCTTT<br>ATTAGCCGTTATGGGATCAGGGGTGCACAAATCCTTCAACATAGGGGACTTGG<br>CTCCATATCATTGCATGGAAGAATTGAGGACAATTACTTTTGAAGAAGCAGAG<br>GATGAAACAGACATGGGGACCCAAGAACAACCACAAGCCTCAGCACAAGACC<br>AGACACAAGCCTCCTCACACAAACAGAACCTTTGGAGCTATTCTTCTGCAGCA<br>AATCTTGAGGTATTAAGTAAGTGAAGGTCTAAGGGCATCCAAACCTCCATAGG<br>GACCCTAAGTAAGTGTGCAAGGCAGGGGGAACCCTCAATCATCCCAAATAACC<br>AAGATACACCCTCTGAAACACAAGTAGACACAAAACAGAACAGGCCTGAGCA<br>ACTGACTGACCAGGGGACCTTGATGAGGGTCCTGAGCCTTAACCTGTGCAGCA<br>CCGGACCTAACCAGACCATATTTGGCATCTCAGTACATGGACCTAGCCACTCTG<br>GGGATAAGACTGCTTTCCACATCCACATGGAAGCACAGAGCCAAGGCAGAAG<br>ATGGCCTGCCAGACAGATTGACAGATTTTCATTCCTTGTTTTG
AmC246/0.011	271	TCTTCTTTTCGTCTTCTGAAACCTCTTCTTTCCATGTTCATTCATCTCATCCTCAAT<br>TGTCTATTTCTTCATGTCGCCTCAGTCCTCCTCTCTTGCTTCATTTTCTCCACAAA<br>TCCCTTAACTATAAAAAGAGCCTAGAAGACTAACACTATTCACAATATACAAA<br>TGAATAGTGTTTTTGCCAACTCATCTGCCACTTCCCACCTGCTACTCCACACCTA<br>CTCCACCTCCCTCGGTTCTCACCTTCCTCCTATTTTCATCTTCTGAATCC
AmC12/0.47	3008	TTTTGAAGTTGAGTGTGATGCATCTGGGGTAGGTATTGGAGGTGTCCTAACTCA<br>AAACAACAAACCTCTTGCTTATTTTAGTGAGAAACTCAATGATGCTAAAAGGA<br>AGTATTCTACTTATGATAAAGAGTTTTATGCTATTATTAGATGTTTAGAACATTG<br>GAGACATTATTTGATTGCAAAAGAATTTGTGTTGCATTCCGATCATTGATGTAG<br>GATTAACTACATCTAATTCTCATGAGCTAACCAAGATCAATACCATGATTCATC<br>ATAGGGAAGACGCTAGAATTCAAGGTCCAAATGTCAAGTCTTTTAATTTTGTAG

		TCATGAACTAAGTCTTCTTTAATTTGAGTCTTTATTTTGTATTTTCATTTTGAAAC<br>AGTCTTAGAGATGATTAAGCCAGGTTTAGGATCAAGCTTAATCATTCTTCATGTT<br>TAGTTAGTCGAGTTGTACTTTTCTTTTTTTGAGTCTAGTATTTTTTTGCCCAAAGTT<br>CCAATGCCATACACGAACAATCAGCCAAGTATAATTCAGAATAATCATGGGAA<br>TATGAAGATCATAAGAAGATAATTGCAAATCACTCAAGGAGCCATTGCAAATC<br>AGCCTAATATTGCTTATGACACTTGGAGCCATTTGAAGATTATTTCTGCCCATAT<br>TGATCTATCCTTAGAAGATCTTTGGAGTATTTTGCTGTTGGAAACCATCCAGAAT<br>ATTGCAACAATATTCTTAAAGGATTTGCTAGCTTATCAAAGGAGTTATTTCAGC<br>CCAAGCAAATATTTCAAGAATAATCTCAATATTTCTAAGAATATAGCTGTTAGT<br>TTTTTATTCTGAATTGTATCTCTTCTTTTGCAAGTTAGTCTTTTAGTTTAAATAGCC<br>TTGTAATTGCTGGTTCATGGAACTGATTTTTGAACATTAATACAAAAAAACCTTT<br>GAGCAAGTTTGCTTTGTTTTGCAATTTATTTTTAACTTGGATTAGTTAAAGTAATT<br>TGAAGTTCCTATGCTTTTCATTCTTTTGGCTAAAGATTGAATGTGTTCTTAGGAG<br>CCCTTAGTTTTAATTCGTGGTGTATCTGTATCGAATTAAACCTTTGGTTCAGCCG<br>TTCTTAGTTGATCTTTTTCAACATATCCCTTTTAATTTTTCTTGTTACAAAACCCA<br>CAAAAATCACAAAAACAAATTCATTGAAACCGAAACAGCAAGTAAACGTGTG<br>CATATAGTTTGGTTATTGTTCGACACGTAGCCAACCTATATCATCTTGGTATCAG<br>AGCCAAGGCTACGCTCCTCGTCTTAATTTCGTTCATTCACAAAGCAAAACACCA<br>TGTCTCAAAGTGGTGAAGAAGAAACAAGTGATATGAGCAGAATTATTGAAGAG<br>CAAAATGCCACCATTAAATATCTTCAAGCCCAACTTAAAGAGTTTCTGCACTTA<br>AAACGCACCCTAAGGGATGGTGGGGATGATGAGTGCGAAAGTCCTAAAGTTA<br>ATAAGCAAACAAGCCGTGGAGATGATCTTAGAGTCGACATTCCAGAATTTGAA<br>GGAAGATTAGATGGAGATGAGTTCCTTGAATGGGTAAGAACCGTTGAAAGAAT<br>GTTTGATTATAAGAATACAGATGAAGAAAAGAAGGTAAAGATAGTAGCATTGA<br>AATTGCGCAAATATGCTTCTACTTGGTGGGCTAGCAAATGTGCAAAACGAGAA<br>AGAGAAGGGAAAGACAAGATTAGATCATGGGAAAAGATGAAAAAACAAATG<br>AAGGAAAAGTTTCTGCCTTCATACTATATGCAAGAGAATTTCACCAAACTTCAA<br>CATCTACAACAAGATGGCAGGAGTGTAGAAGAATATGGTCGAGAATTTGAAAC<br>CATGATCATGAGATGCGATCTTAAGGAGGATGATCATCAAACCTTGGTAAGAT<br>TTTTAAATGGTTTAGATTCAAAAATCAGAAACATTGTTGAATTACAACCTTATTC<br>TTGCCTTGATGATTTAATTAACCTTGCACATAAAGTGGATAAACAATAAAGAGC<br>TAAAATGAAGGAAACATCACGTTTCACTTCATCAAGAACAAACAACTTCAATT<br>CCTACCAAAAGAACACTCCCCATTCCTATAGCCCATTCGCAAGATTCAAAAGC<br>TTTGCAAAACAATCCTTCAAAACAGCCCCTAAACGTGCCCAATGCACACAAAC<br>CGAATACCAATTCATTCACACCTAGAAGATGTTTTAAGTGTCAAGGCCTAGGAC<br>ATATTTCTTCTGAATGCCCAAACCGAAAAATGGTGAACTTGGTTGAGTTTGAAG<br>ACTATCGAGAAGAAAACGAGGAGGATTTCGTTGATAATGAGGAACTTGATGAG<br>GAAATGATATATCCGGATGAAGGAGAACTTCTTGTCATGCGGAGAGCTCTTAGT<br>GGGGTCAAAGCCAAAGATCCCAACCAACAAAGGGAAGCAATATTCCACACAA<br>AATGCACGGTTAAAGGCAAGGTATGTTCTCTTATTATTGATGGAGGAAGTTGCA<br>CTAATGTTGCTTCAAAGACTATGGTGGACAAGCTTAATCTCACCACCACTCCAC<br>ACCCCGAGCCATACATGATTCAATGGTTGAATCAAAACAAAGGTATTCCTGTA<br>AACTCTCAAGTTTTGCTTTCATTTTCAATTGGTAATAGTTATAAGGAGAGTCTTT<br>TATGTGATGTTATACCTATGGATGCATGTCATGTTTTATTAGGAAGACCTTGGCA<br>ATATGATAGAAAAGTTTTGCATGATGGCTTTAAAAATACACATACTTTTGTTTTA<br>AATAACAAGAAGGTCACGTTAGCTCCATATGCACCTGCCCATAATTCTAAATTG<br>CAAGCAACTTTAACAAATTTTCATTCTTTGATTCCTATACTTAAAAGTGAACAA<br>CATGAATTTATGGAACGAAAAGAATTGTTTTTGTTGAATGAAGAAACCGAATCT<br>GTGTTGCATGATCACCCTTTGCTTGTACCTTTGCTTGATGAATTTGCAAAACCTT<br>TCTTGGGTCAATTTGTTGTGGTTTATTTTGATGATAT
AmC32/0.2	4950	TTCATATTTCTAATATGAAGGTTTACCCATGATTATGTATTTTGATACTTAAGTAT<br>CATATAAGTTTAAGCTTTCTAAAAGAAAGTAAGTAATGATGTGTTCATCATGGG<br>AATAATGTTTGAAGTTTAGTAACTTAATCTCCAAGTAAGTATAACAATATGTTA<br>TAGATAGATTTTCATATTATATATGAAGATTGATGAGCATGTAATAATGTTCAA<br>GATTTATTAAGTGGTATTATACTTAATATGATAATTTATTATTGAATTTATTTCAA<br>TAAGTTTACCGAAAATACAATGGTTAAATAATTGTTTGTATTTTCATATGGTATG<br>TTATATCATATATTTTTATAAATTATGATATATTTATTTTGGAATTGTATAAGTAA<br>AGTTGACATAGTAATATGTCATATTCCTTCTATGAAGGAAATGGGGGAGGTAA<br>ATGTTAAATGAATTTTAACATAAAAGTTTAAGGACTAAGAAATAAGTATTTTAT

		TATACTTAAGGTAAAGTCCAAATTTACCCTCATGTCTTCCAATTGTAATTATTTC<br>CTAAATATTTGGAATAATCATAAAATTGGATGTAATTTTGATTTATACAAGATTC<br>AAATTGTTTTATAGTTGTCAAGTAAAGTTTTACTTACAACTTAAAGTTCCTTTATT<br>AGGAAATTATTTGAATAGTCCAAAATGTTTATGTTGTACCATGGATTTAATTAAT<br>TTTATAGTAGAAGTTCTTCAAGTTTGAAGAGGTACTCAAGTATGAGTTTGATCTT<br>TAGTGAATTGGCCATTCTCTATGTTGATCAAAGTATATGTGTTTGTGGGGGAGAT<br>ACCAAGTTTTGATCACCCAAGAACAAACAAAGTTTTAACTTATTCCACTATGAT<br>ATTAATGTTATTTGTCATGATTTAGACAAATATGTAAACTGTATAGTTTAAAATA<br>CTCATCTTTGTGATGTTTAAGCCAAAGTTGTGCACAAAAACAATCTAGTTTGATT<br>GACAAGTGGCTTGTATAAAGTATTGATAGTCGAGTGTAACTTTGATAATGCTAT<br>GACATCTAATTGAGGCAAAGCCGAAAATTAGGGCTTATCAAAGAAGGGATCAC<br>ATTTCTTGACTATGTGAACAAAGTTCAAAGTTAGACAAAGTATTCTTACCAAAG<br>TCTAGGAATTTCATGGTTTAATGAAATTGGTACATGCCCATTCTTCAACACCATG<br>AGTGAAACCCAATTCGACGCTAGAAGAAACATCTAGTCTTGAATTCAATGTGG<br>ATTGATCGCTCATCAGATGTTGGGAAGCAACACTTAAGTGATCCTAAGTTGCTT<br>TATTCGTGATGTTGACAAACGAGGTAGTAGAGTAGTCCCTCGAGGTCTCCATTT<br>TCTAGAGTTATGAAGATCTCTAGATTGAGTGCTTTATTCACGAAAGCATGGTAT<br>ATTAGGGAGAAGCTACCTATATAAGTTCGAGATTTGGCCGTCTTAAAGAAATC<br>AAGGGGCATACTTATGATTTCAATGAACTTATGAATAGATATGGACACATGGC<br>CTTTAACGTGTCAAGGACGTAAGATTAATGAATTATTTCGTGTGTACTACTCCTT<br>CAATTGGTTGGATATATCTGCTTAATTCAATCCGGAAGGACATTAGGGGATATG<br>ATCAATTGTAAAGATGCGTGGTACTAGGTGTCAATTCAATCCGGAAGGACATTG<br>GCATTGATGCATGAAATGGGTTGGAGATTATGGATCGCAAAAGAAGAAAAGG<br>ATTATACACTCTAAATGGGGGAGGATTGTTGGAATTTAGAGTGTATAGTGGTTA<br>TCGGAAAGGTTAATTAATTATATTAAATAAATAATTTAATTAAATTAATGTATA<br>TAATCCCAAGGTTGTAAATTATATGTTTTAATCAAAACATAAAAGTCACGGCTC<br>GTGATTTGGTTCGCGGCTCGCGAAAATACACAAAAAGTTTCTGTTTTCTGGGGC<br>GCACAGGTCGCGGCCCGCGACTAATGTGTTTTTCCGGGAAATTTTCGTGTAACG<br>TTTTGCTTTGTTTTGATGGTTATATAATAACTTCCTACAGTTATTATGGTTATTTTT<br>AGCCATGTTCTTTGGGATGTAAGGGTTGAGTGAAAGGCGACATTGGTTCATGAA<br>TCGATCTCGTGTTTCACGGATCAACCTCGTGTTTCATGAAGCAACACGTTGCTTC<br>ATGAAGTAGTGTGAGCTATTCTATAAATATAGAATGCTTGTGTGTTATTTTACAC<br>ATGCAATTTCTGATTTCTTTTCTTCTCTAGCATTTTACATCGAGAACTTGCAATCC<br>TCGTTTGTAATTTTCTTATCTTCTTCCATTTAAGTTTCTCTTACATCATTCTAATTT<br>CCGTGTGCTCGGAAGTTAGTGTAATTCTTTGTATCTCAGAACATATTTCCGGTTT<br>ATTCCCAAGCACCGTAGTAGGGAGGAAATGATGTTTTAGGAAAGAGCATCTCG<br>CCTAGAATTTATATCAAGTTCCAACAAAATCTTCTTGTTACTATGATCGATATAT<br>GGTGATTCCCGATGATCAAGATTGCATTTGGTGTATGTTAAGCAAAGGTTTGAA<br>CTTGTCATATGTAATTTGGTTTTCTACTACAAACTTGTAACTTTATTTTATATGTT<br>TGATTTATAAAATTAAAGTCACATTTCTAACAATCTAAAACATCATCTTCTAAA<br>GTAAGTATGACAAGTCTCAATGTTTACCACAAACTTTGTCATAATCGACAAATT<br>TGTTGGGAAAGTTTTTAAGTTTGCTTATATGAAAGTTACACCATGATCGGGTGAT<br>GAGATAATCGTAGAGATTTTTGAAATGGTGCATTGATTCGATAAAAGAATGCGT<br>TGGTTCGATAAAGGGACACGTTAGTTCATGAAAGGACACGTTGGTTCATGAAG<br>AGTTGTGGTGTTTCTTGTACTCTTGATCAAGTTATGTAAGAGAGATGGACATAAT<br>TACATGGTAGAGATGATATTTCTCAAGTATGTAAGTTCTCTATGTTAATTTCATA<br>TGTTTATTATTTTTATGATGATAGAGAAGTATAATATGTTACATGTTTAAGCAAG<br>TTCTTATATCCATGGAATTTGGTAAAGTAAGTGATCATGGTTATTTGGTCATGGT<br>TATCATTTGGTGTAAATGATGATACTCCATTTTATTTATCAAAGAAATTTGATGG<br>TTGGAAGTATAAGTAACTTACCATGATTTTTATGATGATTAAAATTATCATCAA<br>AGATGGATAATGTTTGCTATCTTTTGTGTTTACAAGTTTTGTTTTATGCATGATAA<br>GATGATAAATTTCATGGTCATGTTTTGACATGTTTATCTTATTTTATATGTATCAT<br>GTCTTGTTTTATTATAAAAGATGATAAGTGTTATTATCAAGTTGGATAATATTGC<br>TTTTATAGTTCTTTAATGGTAACATGTTAAGTTTTGGGTTTTTGCATGTTTGGTAT<br>GACAAGATAAATTTTGTCATATAAGTTAAACATTATGTGTTTGTTATGTTTGGTT<br>ATCTTTTGACCAAGTTCAATAATGTTTTTGTTATTGATGTATGTTTCACCTTTTGA<br>GTGAATTGTTTTTAAAAGGTTGCATGATCCATGTTTAATTAACAATAAAGTTAAT<br>TAAAATATAAAGTATATTATTGCAATGTTGTTCTTAACAAAAGTTTATGTTGCAT<br>TATTTTGTTTCATGTATGTGTAGAATTATTTCTATGTGTTTGTGCATGATTCAAGT

		TAGTCTTTATGATATTTTCTTTGATTGGTTTATTACCAATGTTGTTTAAATTTGGA<br>CATATAATTTTTATATGGGTTTGTCATATGTATACTTTTACCCAAGGTTATATTAA<br>TTTATTAATATTGGAAAAGGGTACAAAGTTTATGACATGAATAAATTTAAAGGT<br>TTATCATCAAGGATTCATTAAAAGTAAATTTTAATGTAAAGGTGATGTTAAACA<br>AGAATTTGAAAATCTAATAATGTTTTAAATAAAGGTTGAAAGTTGTCAACATTA<br>TTTTAAACTCGGAATTTTATTTATTAAAGTCTTTTATTATGGAGGTTAAGTTCCAT<br>AATGTTAAATAAATAAAAGAGTTTAATGATTATATAAGTATGTTTATATATCAA<br>ATAAATGATAAAGACATATTTGTCTTGTTTTATTTTGATATATATGATGGTTTAA<br>TCTTATATAGTATAAATGTTGATTTGCTAAGTAAAGCAAGATATGAATTCTTTTA<br>TTACTACATTGATTAATGTATTAATTGAAATTCATATGTTACTCTTAATCTAGAG<br>TATATGTCAACACTTTAAGTTTTATTATGGTAATTAAAATTCCATAATATGTTAT<br>TATTTTGATTTTCAAGATGTGCATTATTTGCATGAGTATATGTAAAGTTTTATACT<br>AATATTTATTTTGGAGAAAAGTACTAAATAAGTTTTATTCCGGTTAATGGGTAT<br>GTTAAGGTATCTTATGTTGTCGGATAACGCCTAAATATTTGGTGTGACAATAAA<br>GAAGGATTTAACTATTTAAGTTAAAGTTGATATTAAATGTTTTACTTGGTATAAA<br>GTATTTGTTTTATAGAAAAATCTTAATCATTTTGGTACAAAGGATATTTATTTTG<br>AATTATCTCAAAATTCAAAATAAGGATGCATCTATATAGATTATGTTATTTATA<br>ACATCTACTATGAGTTCTATTTATTAGAACAAGGGATATTATATTTCAATGATAT<br>TTGATTATAATACAAAGTGATGCAATATTTATTTGGAAATTTATTTTCAATGATA<br>GATAGGTTTAAATACCTACAAGTTTGTACAAATGGTTATGACATTTTGATCTGTA<br>ATAGATTGACTTGCTAATCTTTTGATAAAGCATGCATTTCTAAAAGAGAAAAAT<br>ATAAGTTTGTTTTACTAAGGAAAATTTTTAACGAAGTTTGGTTTTGTTAAAGTTT<br>TCGAAGTAAAAGGTTTTTAATATCTTGTA
AmC70/0.1	21	AGGGTTTAGGGTTTAGGGTTT
AmC103/0.062	88	TTTTAGCCCAAGAAGGTGATCTTGGGGCTAAAAATTCTAAGTGTTATCGTTGTC<br>CGCCTCACAAGAAGGAATGTGACTCGGACCCTGT
AmC154/0.027	154	ATTCGGACACCAAGGGAGTCCATCCCAGGGAGGGGCCGAATACCTTCATGTGG<br>CCGAGTACTGGACTGGCCGAATAGGAGGGGCCAACCTCTCCCCTGAGGTGTCC<br>GGCTCCTGTTCTTCATACTTAGGCAAAGTTAGCTAAGTGTTGTGAGGT

AmC5/0.82	4924	ACTTTCCATAACATGATGAAAAGTCGGGGGAAGAATGCATGGAGGGAGGGGG<br>GAGGGAAGCAAGGGGCATAGCAACACAAGCATCAAAGCAAGCATGAACATG<br>AACGACCGGGAGCTTCTCATGATGACAAAGAGGTGTAGGGTGAGACTCAACA<br>GAAGGCACAACAAGGGTATGCACTTGAGACTCCTCAATAAGGGGCAAGTCTAC<br>CTCTAGGTCATCGGTTCCACTAACTAGATGATCGTGCACATATGTGAGGGGTGC<br>ATCATATTGAGGTGCGCAAGGAACCTCATCATGAACCTCATCGGTGAGTGGTG<br>CATCAACACAAGGACATTGATCATCTAGAGGAGAAGGGGAGCTAATGATAGG<br>GTGAGGGGAGTCTAGGTCATGGTAATGGGACTCTAGTCTAGCCTCAAAGACCC<br>TTCTATAAATATTAACGCTCCATATTCTCTCCAACTCGGTCCCTACTTTAACCAA<br>AGGGTCACTAGAAGGGAAGGTCGGTGGAATCTCAAGGAGAGGGAGACAAGGG<br>TACTCTTTCCTATAGAAATGCTTGGCCATCGCTTTAAAGAAGTCCAAACAAATC<br>ACAGGGGTTTGGTCAAGAAACATGTTATCGTGATACTCATCTACGAGTTCCCTA<br>GTCTCACTATTCAAACCTCCGTAAAGATGATGACAAATTTGCCACATTTTAACA<br>TTATACATGCGGAGGAAGAAAGTCTCATACCTAAGAAAGAAGCTCCAAAAAG<br>GTTCATGCTCGAGTTGATAAATGTGGCAATCCATGAAAACAAAAACAAAAATA<br>ACAAAAACAAGAACTATTCACAAAACACACCAAAGCTAAAATCTAAACAAAC<br>GCCTTGTTCCCCGGCAACGGCGCCATTTTGATAACGGGTTTTTAACGCGTCGTTT<br>TACGCTAAGAACCTTGGTTAATCAAACACATTTATAATCACCCAAAATAATACT<br>ACAACGAGTGTAGCGGTAAGTCGGGGGTCGAACCACAAGGAACAAGGGATGT<br>TAGACTAAAGAGTTGGTGTGGGAAAGGTTGATGGTTGGTAGGTAAGAGGATGG<br>GTGAACTAAACTGGGATGAAAACAAACAATGAAAGAAAGCGGGCTTAGGCTA<br>GGTCCACCCTAGGTAGGTCTATTGGAAATAGAGATAGGCTAGGTCGAAGGAGA<br>TCGAACGAAGGTCAATCGAGACACTCTAGATGGCATGGGGAAGTTGATGACCT<br>ATAGTCAATACGAACGACCCATAATCGCCCTATGTCTCACAAGGGAGTGGCAG<br>GACAAGATATGCATCGAATATCAATCAATCAAACCAACATCAACCTCAATCAT<br>CTTCCTAAACATTAAACACAAGACCAAAACCCTAAGGTTTCTCTAACCCAAAA<br>CCCTAAAAGACTACTCTAGCATATTAAAAGCAAAAGCAAGAAGAGAGAAAAG<br>CAACAAAACTATTCTAGAAAGCATAAATGAAATGAAAGACATGAAAGCAAGA<br>AAGAAAGCATAAAGGAATTGAAAGATAAAAAAAACATTAAATCTACACTACA<br>AAAGCAATAAATGAAAGCATTAAAGAAAGCATTAAAGAAAGGAAAGCATTAA<br>ATAAAGAGAACTTACATAAATGAGAGTGTAGAAATGAAGAACAAAACATAAA<br>GGAAAGCATAAAACTAATCTAGGGCAAAAATGAAGAAGAAAAGCATTAAATG<br>TTGATGAACAAGAATGGGAGGGTTACTCCCAATCAAGTCATAGGGTTTATGTTC<br>AGAAAAGTGCCTTTATATAGGCTACAAGAGATGGGAACAAAAGCCCTAAAAG<br>AATCCTACGTGGCAAGCTTCGAAGAGAAATCGAAATCGCGAGTTTCTGAGTCG<br>CATTCGACCGAATGCAGTGAAGATTCGCCCGAATCTAGTCAGGTTCGACCGAA<br>CCTAGGTCGAATTCGACCGAATCTGGGCTGTTCTGGGCTTCTGATGAGCTGCAG<br>TGTGAAGCTTCGACCTGGCTTCGACCTGGCTTCGACCTCAATTCGACCGAATCC<br>AAGGAGCTTCGACCGAATCTTGGTGATTCTTGGCTTCCAGAGACTTTGAGCATT<br>GGGCTTGGGCATGGCATGGTCCATTCGACCGAATGGACTTCTCATTCGACCGAA<br>TGTGGTCATTTTGGCCACATTGAAGCTTCAAACTATCATCAAACGACTTCCGAT<br>CTTTGCGATACTTTCGGGGATGACCCGAAACACTTGTGGGAGCCTCGAAACGC<br>GTGCCTCTTCTTCGACTTTGATGTCTAAGCCTCGAACTCGATCCGAATTACCTCG<br>AAATCTCCCGAAAACACCTGAAATGACCTCTAAACACCATGGAGACAAGAAC<br>AAGGTAAAATCGAGTGTAAAACGCATAAAACCAATGTAAAACATCGAGACTC<br>GACACCAAAGTAGGGATATAAATGTGCTCTAAATCGAGCACATCAACACCCCC<br>AAGCTTAACCTTTGCTAGTCCCTAGCAAAGAGACTAGGTAGGATAACATGAAA<br>GCACAAGGGCATCCCAAAAACAAACTAACTACAAAAGCAAAACAAAACTAA<br>ACTATGAAAGCAATAAAACCTAAAACTCACTGTCGTAGGAATCACGGTCGCAC<br>TTAGCACGTACGACAAGCCCTTTAAACCTCAAGGTCTCCCAAAGAGGACGAGT<br>AAGGTCTCGTGAGGGTTTTCAGAAGGAGACCCACAAATCCAAAGCAAACAAA<br>CATGGTGAGCGGAGAACAAAACAATACAAGCATGTGCATGAGCAAAACTCTT<br>CCCTAGCCATAGGTGTGCAAGTACAATGGTCTCAAAGAGCGCAATACCAAAGA<br>GAATGACCAATTCCACAAACTTGAGATCACTAGGGTAAGCTACATCACAGGCT<br>ATGCCACCCACTACCTAGAGAGGTCACAAGTCTACGCTCCCCGGGTAATTTCTC<br>AAAAGATGTGAGAAATATGCAATGACGCTCATGTGTTTGATTATGGTTCGTGTT<br>TCGCTCATAATCTTCCGACTAAAGATGAGTCGGGAATCAAACACCTAATAGGG<br>GAGAATCATGGGGATTGTGTCATATCTAACTCCAGCACAGAACAAACTAATCG<br>GACCAAGTCGAACAGGTTAGCAAAGGGTTGTAACGTTGGCTTAGGGTTAAGGT

		GGGATACATTTGGGAATGTGGAGCTAAGTAGAGGATAGCAAGTGACCCTAAAC<br>AAAACACCCACAAACATCTCATGATAAACCAATGAAAACTATATCCAACAAC<br>CATCAATAACCCAATAACCTCATATAAGCACAATGATCTCTATCATAAAACAC<br>CATCAAAACATGTAAAATCTCATAAACACAAGTGTTTCCAAGCTCATCTCCCAA<br>TAATATAACGACCTTCTCACTCACATGAATATGTATGTGTATAAAAACTCAATG<br>AAAAACAAGAGAGAGGTCTCCTCAAAACATAGAATGTGTATGAATAATAACA<br>ATGAAAGCACTAGAGGAAATAGGAGAGAAATAGTATGAGCTGGAAATGAAAT<br>GAGTAGAGTCATGAAAACCAACAACCAACAACATAACCAATAACCCACAAAT<br>CACTGTAACACACAACAAACCCCACCATCACTAAGCTATCCTCTAGGTAAGAA<br>GAGGGAACAATTTGGCTCATGTGGAGGCTAAATAATGTGGCTACAAAAGAAA<br>AGGAAAAGGCAAAAACTTGGCTTATCCTAAAGTCAATTGCGTTCAAGGTAGGC<br>TATTTGGCTATGTAGCTAAATTCTAGCGAACGTGTTATCATCCCTGGTCTCAAGA<br>AAAGCCGACTATACTGGAGAAGAACCGACACAATACTTAGATCCCCTAACTAG<br>CACTCAAAATGAATGGAACTCGCAAGGAGTCCAAGAAGGCTCAATCCTCACA<br>GGCTAGTCGAAATGCGCCTCTAAAGGTAGTCATCCAAGCCACCAACCAAGCAA<br>ACCCCAATTCCCCCAAGCTATGTGGAAAATAGGTCAGACTCTAGGTCCCAAAA<br>CATGCACAGGGCTAAGGAAATACGAGTTTGCATAAGGTAAGACCACAGAAGT<br>ACACACCAGAAGCAAGCATGCCAGTTTCACAACTCAACCATCTTATTGAAAAC<br>TAAACATGAACATGGAATATATACACTATGTATGAATGCAAGATGAATATGCG<br>AAAAATATACAAAATAACAAATGCAATGAACTAAACTATAGCTATATGAAACT<br>AAATGCACACACAAATGGGTAGAGCGACAATGGAGTTGACCATCCCACCCCC<br>AAGCTAAAGCTTTGCTAGCCTCTAGCAAAGAAGGAGAGAAGAAAGATGAAAC<br>ATTAAAAACCATGGTCAACAAGATTGTCGCAACACCCAACACAAACACAAGC<br>TAGCCAATAATAAAGAGAGAAGAAGGTCATACCTAGGCTAGGAGGGCATTCA<br>CTCCTAGCATTTCCATCACTATGAGGTGGTCTAAGAACTCTCCGCTCCTCGAAA<br>GAAATGGAGGCATGAAGCACGAAGCTAGAGCAATTGCCTCCCCCAAGCTAGG<br>GTGAAGCTATATTCTTCATGTTTGGCTCATACGCTCTTTTGAGCCTTCTTTCTCTT<br>ACTCACCCTTGCGGGACCGTTAGCATTGACCCTCTCAAAATCATCAAATAAAAC<br>ACAAGCAAACTTGTGGAGGGTCAATTCTAATGGTCGTTTGGGTCTGCGAATACT<br>AACAAACAACACTACAAACCAAACGTGAGCAATGACTAGCAAAAATGCAATG<br>CAATGAATGAAAAACAAAAATGAAACAAAAACAATATCTACAGTTGGGTTGC<br>CTCCCAACAAGCGCTTCTTTTAGGTCTTTAGCTAGACCCGACTCACTACGGGGT<br>AGCGAGTCAAACTCCGTAGGAGTTTATCAAACTCACTCCCCACAACAGCAGAA<br>GTTGGGGGGTGAAAGAGGGAGAGAAGAAAGAAAGGGGGAAACAAAACAAAA<br>ATCG

Supplementary Table S10. High confident putative satellite DNA repeats identified in the genome of *Amaranthus hypochondriacus* using RepeatExplorer2/ TAREAN.

**Tandem Repeat/ Genome Proportion, %**	**Repeat Length, bp**	**BLAST Similarity**
AmH4/1.5	42	100%/100% of coverage/identity with AmC9 in *A. cruentus*<br><br>100%/91% of coverage/identity with AmC27 in *A. cruentus*<br><br>60%/96% of coverage/identity with AmT2 in *A. tricolor*
AmH51/0.14	169	98%/85% of coverage/identity with AmH9 in *A. hypochondriacus*<br><br>99%/85% of coverage/identity with AmC4 in *A. cruentus*<br><br>100%/85% of coverage/identity with AmT1 in *A. tricolor*<br><br>100%/73% of coverage/identity with AmT129 in *A. tricolor*

Supplementary Table S11. Low confident putative satellite DNA repeats identified in the genome of *Amaranthus hypochondriacus* using RepeatExplorer2/ TAREAN

Tandem Repeat/ Genome Proportion, %	Repeat Length, bp	BLAST Similarity
AmH9/0.45	169	98%/85% of coverage/identity with AmH51 in *A. hypochondriacus*<br><br>100%/98% of coverage/identity with AmC4 in *A. cruentus*<br><br>100%/90% of coverage/identity with AmT1 in *A. tricolor*<br><br>98%/77% of coverage/identity with AmT129 in *A. tricolor*
AmH27/0.24	4133	13%/72% of coverage/identity with AmH6 in *A. hypochondriacus*<br><br>99%/96% of coverage/identity with AmC32 in *A. cruentus*<br><br>*Amaranthus palmeri* clone NJ_Ap_14, NJ_Ap_15, complete sequence
AmH176/0.021	413	No results
AmH210/0.015	1194	*Amaranthus hypochondriacus* mitochondrion, complete genome<br><br>*Amaranthus retroflexus* mitochondrion, complete genome<br><br>*Amaranthus tricolor* chromosome 1 mitochondrion, complete sequence<br><br>*Amaranthus tricolor* mitochondrion, complete sequence
AmH209/0.016	565	No results
AmH218/0.015	1061	No results

Supplementary Table S12. Putative LTR repeats identified in the genome of *Amaranthus hypochondriacus* using RepeatExplorer2/ TAREAN/ DANTE_LTR.

Tandem Repeat/ Genome Proportion, %	Repeat Length, bp	DANTE_LTR Results	BLAST Similarity
AmH3/1.6	5302	Class_I| LTR| Ty1 Copia| SIRE	No results
AmH6/0.57	5312	Class_I| LTR| Ty1 Copia| TAR	10%/71% coverage/identity with AmH27 in *A. hypochondriacus*<br><br>12%/73% of coverage/identity with AmC32 in *A. cruentus*
AmH26/0.24	3949	Class_I|LTR|Ty3 Gypsy| chromovirus|CRM	32.5%/93% of coverage/identity with AmC12 in *A. cruentus*

Supplementary Table S13. Sequences of the DNA repeats identified in the genome of *Amaranthus hypochondriacus* using RepeatExplorer2.

Tandem Repeat/ Genome Proportion, %	Repeat Length, bp	Sequence

AmH4/1.5	42	GATGAACAATGAACAAGGATCAATGATCAATGAACAATGAAC
AmH51/0.14	169	GTTAGAATTGAAAATATTTGTCATATGGTTCAAATTGGGTATTAAGTTGCGTTTT<br>TATTGTTTTTTAAGCTTTTTTGGCACTTTCGCGCGTAAAGTAGCTCAAACGTGAT<br>TTATTACGCATGAAACTTGACACACATCACTAATGGGTATATATTATTGTGTTG<br>AAGTG
AmH3/1.6	5302	GAATTGATAAATCTGATATAAGTGCTAATGGAGACAGTGCAGTGTTCCCAACG<br>AAACAGGTCTGGAGAAGTTGGTCAACTAAAAGAACGTAGGAAAAATTAGTTTT<br>TCTTTATTTAGTTGATTGTACGGTTAATACTAAATAATAGCATAAGAGATTAATT<br>GGCAAAATTAATTAAAATTAATCTAAGGGTTAAGAATAAATCTCCTTATTTCTC<br>TCCACAAAAATAGGGAAGAAGTTAGGGTTTAGTTGAATTAGTCTTCATTTACTA<br>TTTTTAGCCAAATTAACCGTGGGACTTGGAGTAACCTCCAAGCTTCCCACTTCCT<br>CTACTCCCATCACTTGGGATCATGGAATTATGGGTTTAAGAGTCTCATGATTAT<br>ATATATATAATATTTTGCAACCGGCACAAATTTGGTGATCAATAGAGAGAAAA<br>ATATTTTTCCGAAAATATTTGTGCCCAAAATTTCGTGAGTTTTCTAAATCAAGAA<br>AAGGAGATCTTGAAGCTTTTAATTGCTAATTAATTCCAAGATCAAATCCTTGTG<br>CTATTCTTGTTTTGTTCATCCATTTTAGAGAAATTTTGTACGGGTTTATTTGAGAG<br>AATTTGTAATTAGACTTGGGTAAGTCTAAAGGGGAATAGAGAAAGAAAATTGA<br>GAAAGAGATAAAGAAAGAGAACGAGAAGGAGAAACCGAAAAGAGAAAGAG<br>AAGAAGAGAAGGAGAAGAAATTATTTTATGTAATAGAGAATTGACTTACATAA<br>AATAATAGAGAAGAGAAATCCCTAGTGGGTCGTGGTTCTTTTTCGGCCTAGAAA<br>GTTTCCACGTTAAAATCTTTGTGTTCCTTTATAGTTTTTTTTATTTGCTTTGAGTAT<br>TTTTTATTATTTAAATTCCGCAAGAAAACAGGGCAAAAATACTCTAAAGGCAA<br>CACAACAATTCACCCCCCCTCTTGTTGCAGTTCATTGTCTCTAAGTGTTTCTACA<br>ATTGGTATCAGAGCCCTGTTCCTCATTTGATCAGGAAACCCTGAGAGCCGTATC<br>TTGGTGTATTAGAGATGTTGAAAATGAATGAACGTATGGAAGAAGGTTACTCTA<br>CTCAGAGACCACCAATGTTCGATGGAAAATTCTACACATATTGGAAGAATAGG<br>ATGGAGATCTTCATCAAAGCCGAAAACTATCAAGTTTGGAGAGTCATCGAAGT<br>TGGAGATTTTGAGGTAACAACTACCAACTCCAATAACGAGACTGTTCCCAAAC<br>CGATAACAGAATATGAGAAAGAAGATTTTCAGAAAATGGAGATGAATGCTCTT<br>GCCATCAAATTACTTCACTGTGGGCTTGGACCAAATGAACACAATCGCATTATG<br>GGTTGCAAAACAGCAAAACAGATTTGGGATCTGCTGGAAGTTACCCATGAAGG<br>AACGAGTGAAGTAAAGCGTTCCAAGATTGACTTGCTCATGTCAAAATATGAAA<br>GATTTGTTATGGAACCTAGAGAAAGTATTCAAGAAATGTTCACTAGGTTCACGA<br>ACATCACAAACGAACTTGTCTCTCTTGGAAGGCTTATTCCCTCAGATGAACAGG<br>TAAGGAAAATCCTAAGAAGTCTACCTCAAGATGATCGCTGGAGAGCTAAGGTC<br>ACAGCCATACAAGAATCCAAAGATTTCACAAAGTTCAATCTGGAAGAGTTGGC<br>TGGTTCCCTTATGACTCATGAATTACATCTGGGAACAGCTGACAGTTCCAGAAA<br>CAAAGGCTTGGCTCTGGCAGCGAATGAACAAGAAGAGTCAGAATGTGATGAG<br>GAAGAGGCAGCAATGTTGGTTCGAAAATTCAAGAAGTTCTTCAGGAACAACAG<br>ATACAACAATCAAAGAAACAACAAAGAAAGAGGAACTTCAAATTCCAAAACA<br>TATGAATGCCACAAATGTGGAAGTACCGAGCACTTCATCAAGGAATGCCCACT<br>ATGGAAGAACGAGAAGGGCAAAGGGAAGGCAAGGGAAACAGGAAGACAACC<br>AATAAAAGGAAACTTCAACAAAACTGACTTTCGCAAAGCCATGATAGCTGCAT<br>GGGGAGAATCCGAAAGCGAGGCCGAAACCGAAGCTCCCGTTGAAGAAGAAGA<br>AGCAGCCAATCTCTGTCTCATGGCCACGCATGATGAAAAGTCTAAAGGAAAAG<br>AGGTACTGTCTTCTAATTCATTTCCTAACCATTTATTTAGATTGGATAAACATGA<br>ATTAATTAAAATGATTTTAGAATCTCACGAAAATTTAGAAAAACAAACAGCCA<br>TCTGTCTCAAAACAGAAAAAGACCTTAAGGCTTGTAAAGACCAAATATCCTAC<br>CTTAATTCTTTTAGGTCCGATGTGCAAACTCGATTCTTTGAATTACTTGATAAAA<br>ATGTGATGTTAAAAGAAGCTATAGAAAAGGTTAAACAAGAAAACATCATGTTA<br>AATATTGAATTAAATCAACTTAAGTTGCTAGAAATAGAATTGAGTAATGAGTCT<br>AATAAAGAAATTAAATGTGTTTTTAATCAAATGAAATTAGACCTAGAATCCGC<br>CATAAATGAAAATGAACAACTTAAGCTTGAATTAAATTCAAGAGAAAAAGGG<br>AAAATCAAAGAAGTTCCCAAATGGATTCTTAATGCTAAAACTAAAGGAACCGA<br>AGGTTTAGGATACAATAAATATGATAAAAAGAAAAAGGTCTATGTTGATCTCC

		CAAGTAGTAAGGTCTGTTCCTTTTGTGGCAAAACCGGGCACCTGAAACATCAAT<br>GTTTAAAAAGGGAACAGCACAACAAAACGAACCAAATATATGTCGATAGAGT<br>TTGGATTAAGAAATATGATTCATGTATAATCGACATGGAACCCAAGGATGGCT<br>GGGTTCCACCCTCTAACAACTAAGTTCGTTTTGCAGGTTCTAGTGAGGGGGAAC<br>AGCTCATGGTATCTCGACAGTGGCTGTTCCAAGCACATGACAGGCGATAGATC<br>AAAATTTCTCTCACTAGAAGCCTATGATGGAGGAACTGTGACCTTTGGCGACAA<br>TATGAAAGGAGAAATCATTGCCAAAGGAAAGATAGGAAGGTCAAGTTCCCAT<br>GCAATAGATAATGTATTTTTAGTCGAGAATTTAAAACACAATCTTTTAAGTATC<br>TCTCAATTTTGCGACAAAGGTAACTCTGTAAACTTTACTTCTGAAAAGTGCATC<br>ATTTCTAGGAACGACACAGGAGACACCATTCTGGAAGGAATCAGAAAAGGGA<br>ACACCTATGTGGTGGACCTGCACACTGTTCCTAAAACCAGTTTAACATGTCTGA<br>GTGTTATAGAAGATGACCCTCTTCTTTGGCATAAACGATTAGGTCATGCTAGTTT<br>TTCATTGCTTAATACTTTACGATCTAAAGACCTAGTAAGAGGATTACCGTCCCTT<br>AAATTTCAAAAGAATGAAGTATGTGATCCTTGTGCCAAAGGAAAACATGTAAG<br>GTCATCCTTCAAACCTAAAAATGTCGTGACCACCTCAAAGCCACTTGAATTAAT<br>TCATATGGATCTTTGTGGACCAATGAGAATCCAAAGTCGCAGTGGTAAGCGAT<br>ATGTATTTGTTATAGTTGATGATTACAGTAGATTTACATGGACTTTATTTTTATCT<br>AGCAAAGATGAGGCTTTTGATGAATTTGTTGCATTTGCCACTAAAATTCAAAAA<br>TCTAGTAACAATCAACTTGTCCACATAAGATCGGATCATGGTAAAGAATTTGA<br>AAATTCAAGGTTTATGGATTATTGTAATGAGCATGGTATAAGTCACAATTTTTCC<br>GCACCTAGAACACCACAACAAAATGGTGTGGTTGAACGTAAAAATAGAACCTT<br>AGAGGAAATGGCTAGAACCATGTTAATAGCTAGTGGTCTACCTAGAAATTTTTG<br>GGCCGAAGCCGTTAATACTGCATGCTATATATTAAATCGTGTGTTAATTAGACC<br>AATCACTTCTAAAACCCCCTATGAACTATTTAAAGGAATTAAACCTAATATTTC<br>ATATTTTCGAGTCTTTGGATGTCGATGTTTTGTGCATGTAAATGGAAAACGAAA<br>TATAGGTAAATTTGATGAAAGAAGTGATGAAGCAGTATTTCTTGGCTATTCATC<br>TCACAGTAAAGCTTATAGAGTTTATAATAAGAAAACTATGTGTGTTGAAGAATC<br>TGTTCACATTATTTTTGATGAAACAAATTTTATGACAAGTGAACAAGAAACAGG<br>TGATTTTAAGATAGGTCTTGCTAATCTGGAAGATGATGAAAACATACATAAGG<br>AGCAAGATCAAGAAGAACAACAACAGGTTCAGAATGCTGAACCAAATCAAGA<br>ACAGGAACAACAGGCAGATCAGGAACAGCCTGCCGTTCCTAATCCAGATATTC<br>CTAGAGAAGTTCAAGAAGAAAGAATTGAAGCTGAACCAAATCAGAATCAAGT<br>CAGTGAGCCTGCTTCCACATCTGTCTCCACAAGAGACTTTGTCCCAAAGCCGTG<br>GAAACATCAAAAGTCACATCCACTAGACTTGATTCTAAGTGACATAAGCAAAG<br>GCATACAAACAAGATCCCAAATGAGAAACTTCTGTGCACACTTTGCATTTCTCT<br>CAACATTGGAACCCAAGAATCATGAAGAAGCATTACAAGATTCAGATTGGGTT<br>GTTGCTATGCAAGATGAATTAAATGAGTTTGAAAGAAATAAAGTGTGGCACTT<br>GGAACCAAAACCCAAGAACAAAAAGATAATTGGTTTAAAATGGGTTTTTAGAA<br>ACAAGCTGGACGAGCATGGCACAATAGTAAGAAACAAAGCAAGGTTGGTGGT<br>AAAAGGATACAATCAACAGGAAGGTATTGACTATACTGAAACTTTTGCTCCAG<br>TAGCTAGATTAGAAGCTATTAGAATTCTTATTTCATTTGCTGCTTTTATGAATTTT<br>AAATTATATCAAATGGATGTGAAATGTGCATTCTTAAATGGTTTTCTAGAAGAA<br>GAAGTCTTTGTGGAACAGCCACCTGGCTTTGAAAACCCAACTTCTCCGGATCAT<br>GTCTATAAGCTTGATAAAGCTCTTTATGGGCTAAAACAAGCTCCTAGACAATGG<br>TACGAGAGATTATCTAAATTTTTGATTGAAAATAAGTTCATAAGAGGTAAAATT<br>GATAAAACCTTATTCTTTAAGAATAAAGGTTCGGATATTTTAGTTGTTCAAATAT<br>ATGTTGATGATATTATTTTTGGAGCCACTAATGAATCGTTATGTAAAGAATTTGC<br>TGACCTAATGAGTGATGAATTTGAAATGAGTATGATGGGAGAATTAAATTTTTT<br>TCCTTGGTTTGCAAATTTGCTAACCTAAAGTGAAATTGATCTCTACTGTTCCAA
AmH9/0.45	169	GTAGTTAGAATCGAAAATAATTGTCATATGCTTGAAATTAAGTGTTAAGTTGCG<br>TTTTTAACGGTTTTGAAGGATTTTTGTCACTTTCGCGCGTAAAATAGCTTAAACT<br>TGGTTTGTTATGCACGAAACTTGGCACACAACACTATTTGGTATATATTATTGTG<br>TTGAA
AmH27/0.24	4133	ATAGTATGGTAACATGTTAAGTTTTGGGCTTTTGCATGTTTGGTATGACAAGATA<br>AATTTTGTCATATAAGTTAAACATTATGTGTTTGTTATGTTTGGTTATCTTTTGAC<br>CAAGTTCAATAATGTTTTTGTTATTGATGTATGTTTCACCTTTTGAGTGAATTGAT<br>TTAAAAGGTTGCATGACCAATGTTTATTTAACAATAAAGTTAATTAAATTATAA<br>AGTATATTATTGCAATGTTGTTCTTAACAAAAGTTTATGTTGCATTATTTTGTTTC

		ATGTATGTGTAGAATTATTTCTATGTAATTGTGCATGATTCAAGTTAGTCTTTAT<br>GATATATTCTTTGATTGGTTTATTACCAATGTTGTTTAAATTTGGACATATAATTT<br>TATATGGGGTTGTCATATGTATACTTTTACCCAAGGTTATATTAATTTATTAATA<br>TTGGAAAATGGTACAAAGTTTATGACATGAATAAATTTAAAAGGTTTATCATCA<br>AGGATTCATTAAAAGTAATTTTAATGTAAAGGTGATGTTAAACAAGAATTTGAA<br>AATCTAATAATGTTTCTAAATAAAGGTTGAAAGTTGTCAACATTATTTTAAACT<br>CGGAATTTTATTTATTAAAGTCTTTTATTATGGAGGTTAAGTTCCATAATGTTAA<br>ATAAATAAAAGGAGTTTAATGATTATATAAGTGATTTTATATATCAAATAAATG<br>ATAAAGACAAGTTTGTCTAGTTTATTTTGATATATATGATGGTTTAATCTTATAT<br>AGTATAAATGTTGATTTGCTAAGTAAAGCAAGATATGAATTCTTTTATTATTACA<br>TTGATTAATGTATTAATTGAAATTCATATGTTACTCTTAATCTAGAGTATATGTC<br>AACGCTTTAAGTTTTATTATGGTAATTAAAATTCCATAATATGTTATTATTTTGAT<br>TTTCAAGATGTGCATTATTTGCATGAGTATAATACTAATATTTATTTTGGAGAAA<br>AGTACTAAATAAGTTTTATTCCGGTTAATGGGTATGTTAAGGTATCTTATGTTGT<br>CGGATAACGCCTAAATAAATTTGGTGTGACAATAAAGAAGGATTTAACTATTT<br>AAGTTAAAGTTGATATTAAATATTTTACTTGGTTTAAAGTATTTGTTTTATAGAA<br>AAATCTTAATCATTTTGGTACAAAGGATATTTATTTTGAATTATCTCAAGATTCA<br>AAATAAGGATGCATCTATATAGATTATGTTATTTATAACATCTACTATGAGTTCT<br>ATTTATTAGAACAAGAGATATTATATATCAATGATATTTGATTATAATACAAAG<br>TGATGCAATATTTATTTGAAAATTTATTTTCAATGATAGATAGGTTTGAATACCT<br>ACAAGTTTGTACAAATGGTTATGACATTTTGATCCTTTAAGAATGATTTGCTAAT<br>CTTTTGATAAAGCATGTATTTCTAAAGGAGAAAAATATAAGTTTGTTTTATTAA<br>GGAAAAGTTTTTAACGAAGTTTGGTTTTGTTAAAGTTTTCGAAGTAAAAGGTTTT<br>TAATATCTTGTATTCATATTTCTAATATGAAGGTTTACCCATGATTATGTATTTTG<br>ATACTTAAGTATCATATAAGTTTAAGCTTTCTAAAAGAAAGTAAGTAATGATGT<br>GTTCATCATGGGAATAATATTTGAAGTTTAGTAACTTAATCTCCAAGTAAGTAT<br>AACAATATGTTATAGATAGATTTTCATATTATATATGAAGATTGATGAGCATGT<br>AATAATGTTCAAGATTTATTAAGTGGTATTATACTTAATATGATAATTTATTATT<br>GAATTTATTTCAATAAGTTTACCGAAAATACAATGGTTAAATAATTGTTTGTATT<br>TTCATATGGTATGTTATATCATATATTTTTATAAATTATGATATATTTATTTTGGA<br>ATTGTATAAGTAAAGTTGACAAAGTAATATGTCATATTCCTTCTATGAAGGAAA<br>TGGGGGAGGTAAATGTTAAATGAATTTTAACATAAAAGTTTAAGGACTAAGAA<br>ATAAGTATTTTATTATACTTAAGGTTAAGTATTTTATTATACAGGTAGTAGAGTA<br>GTCTCTCGAGGTCTCCATTTTCTAGAGTTATGAAGATCTCTAGATTGAGTGCTTT<br>ATTCACGAAAGCATGGTATATTAGGGAGAAGCTACCTATATAAGTTCAAGATTT<br>GGCCGTCTTAAAGAAATCAAGGGGCATACTTATGATTTCAATGAACTTATGAAT<br>AGATATGGACACATGGCCTTTAACGTGTCAAGGATGTAAGATTAATGAATTATT<br>TCGTGTGTACTACTCCTTCAATTGGTTGGATATATCTGCTTAATTCAATCCGGAA<br>GGACATTAGGGGATATGATCAATTGTAAAGATGCGTGGTACTAGGTGTCAATTC<br>AATTCGTAAGAACATTGGCATTGATGCATGAAATGGGTTGGAGATTATGGATC<br>GCGAAAGAAGAAAAGGATTATACACTCTAAATGGGGGAGGATTGTTGGAATTT<br>AGAGTGTATAGTGGTTATCGGAAAGGTTAATTAATTATATTAAATAAATAAATT<br>TAATTAAATTAATTAAGTATATCCCAAGGTTGTAAATTATAGTGGTTTTAGTCAA<br>AACATAAAAGTCACGGCTCGTGATTTGGTTCGCGGATCGCGAACAGCAGGCAG<br>AATGCTACTGTTTTTTTGGAAATCGAAAGTCGCGGCCCGCGAAAAACGTGTTTT<br>TCTGGAAATTTTCCGTGTAACGTTTTGCTTTGTTTTGATGGTTATATAATAACTTC<br>CTACAGTTATTATGGTTACTTTTAGCCATGTTCTTTGGGATGTAAGGGTTGAGTG<br>AAAGGCGACATGGGTTCATGAATCGATCTCGTGTTTCACGGATCAACCTCGTGT<br>TTCATGAAGCGATACCTTGCTTCATGAAGTAGTGTGAGCTATTCTATAAATATA<br>GAAGGCTTGTGTGTTATTTGACACATGCAATTTCTGATTTCTTTTCTTCTCTAGCA<br>TTTTACATCGAGAACTTGCAATCCTCGTTTGTAATTTTCTTATCTTCTTCCACTTA<br>AGTTTCTCTTACATCATTCTAATTTCCGTGTGCTCGGAAGTTAGTGTAATTCTTTG<br>TATCTCAGAACATATTTCCGGTTTATTCCCAAGCACCGTAGTAGGGAGGAAATG<br>ATGTTTTAGGAAAGAGCATCTCGCCTAGAATTTATATCAAGATCCAACAAAATC<br>TTCTTGTTACTATGATCGATATATGGTGATTCCCGATGATCAAGATTGCATTTGG<br>TGTATGTTAAGCAAAGGTTTGAACTTGTCATATGTAATTTGGTTTTCTACTACAA<br>ACTTGTAACTTTATTTTATATGTTTGATTTATAAAATTAAAGTCACATTTCTAAC<br>AATCTAAAACATCATCTTCTAAAGTAAGTATGGCAAGTCTCAATGTTTAACCAC<br>AAACTTTGTCATGGTTGACAAATTTGTTGGGAAAGTTTTTAAGTTTGCTTATATG

		AAAGTTACACCATGATCGGGTGATGAGATAATCGTAGAGATTTTTGAAATGGT<br>GCATTGATTCGATAAAAGAATGTGTTGGTTCGATAAAGGGACACGTTAGTTCAT<br>GAAAGGACACGTTGGTTCATGAAGAGTTGTGGTGTTTCTTGTACTCTTGATCAA<br>GTTATGTAAGAGAGATGGACATAATTACATGGTAGAGATGATATTTCTCAAGTA<br>TGTAAGTTCTCTATGTTAATTTCATATGTTTATTATTTTTATGATGATAGAGAAGT<br>ATAATATGTTACATGTTTATGCAAGTTCTTATATCCATGGAATTTGGTAAAATAA<br>GTGATCATGGTTATTTGGTCATGGTTATCATTTGGTGTAAATGATGATACTCCAT<br>TTTTATTTATCAAAGAAATTTGATGGTTGGAAGTATAAGTAACTTACCATGATTT<br>TTATGATGATTAAAATTATCATCAAAGATGGATAATGTTTGCTATCTTTTGTATT<br>TACAAGTATTGTTTTATGCATGATAAGATTATAAATTTCATGGTCATGTTTTTGA<br>CATGTTTATCTTATTTTATTTGTATCATGTTTTGTTTTATTATAGAAAGATAATAC<br>GTATTATTATCAAGTTGAATATAATTGCTTTT
AmH176/0.021	413	AGCTATGCAAATTGGTGGCATAGACCTATCGAGTTTCAAGTTGATGATAAGGTT<br>TTCCTTCGTGTTTCACCAACTAGAGCAGTAAAGAGATTCTTGAAAAAGGGTAAA<br>TTAGTCTGAGGTTCGTGGGAACCTAGGAGACTTTAAAGCGGATTGAAGAAGTA<br>GATTATTCACAATGATAATGATGAACATTGATAGTTTTGTGACATAACATAGGA<br>GTTCGTGGAGGTAAAGTATATCATG<br>TCATTAGTGTTAGTAAAGCATAGAAATGAACATAAGAATAAGATTAGAGCTTA<br>TAGTTTGAGTTGCTGAAAAGTGTTCAGACTTCGTGAAAAAAATAATGTTGACTT<br>GTAGGAGTAAATAGTAAGTTTTTTAAAAGTTTGAGATGAGATTCAAGTTCTTT<br>GAATAGACAAAAG
AmH209/0.016	565	ATCTTACTATTTATTAATGAACAATTGTTGTTAAGCTGCTACCACGTGTTGGTTA<br>ATCGATTTAGATTCTTCCACGTTTTGGGTTTCCTCTTTTCATTACGTCACTTTCTCC<br>CCACCAATTCACTCACATTCCCTCACTTTCCATATCTGACTCCTCACTTCATTCA<br>CCTTCCTTTTCTGAATTTTCATCTACCCTCATCTCTTCTTGAATTGTCACTTTCTTC<br>ATGTCGCCCATTGTTGAGCTTCTTCGGTCCTTTATTTGTTCATTTCTAGGCTGCAT<br>TTCAGGTAATTGAGAACTTTCAGATTATAATTTCATTTGGATTAAGGGATGAAA<br>AACTTAGGGTTTTGTTTATTTTTTGTTTGGTTCGATGGTATTTGTTGGTTGATGTA<br>GAATTACTGCTATTTATGATCATGAATAGTATCTTAATACCCAGTTTTTGTATTG<br>GTGGCTCAATTGTTTTAAAACGTTTCTGATTATTGGTTTATAGATAAGGGATTTG<br>GATTGTTTAGATTTTTCTAGGGTTAATTTGTTGAGAATCTATTACATAAAAAAAG<br>AAAGCAAAAA
AmH210/0.015	1194	CTCGGTAGAGTGAATTTAAAAAAATTAGGAATTAGCCTCAGAGAATCTTATATT<br>CATAATAGTGATATAGTCAAAATATATTCATATGCTGAATTGTTAGAAATTTTA<br>CATAAGGATGTTAACCGGGCTCAGTATGAGAGTATGTTATTAAACATAGCTGA<br>AGCAAAAAGACAGATCATCATTAATTTCTGGGATAAATTCTACCAAGAGAATC<br>TTTCTTCAAATAATGATGGCGATGAATCTAAATCTAACAGAGCAATGTATAAAG<br>AAGAAGTATTTCAAATATTGAATGATTTCAAATTAAATGAAACAAAGCCATAT<br>AATGATAAAAAATTAAGAGATTTGCAAGAGAAGATTGAAAATCTTACAATGAA<br>GTTTGATGAAAAATCAATTAATAAATCATGCAATCTCATTATGAGTCTAATGAT<br>TAGACCTGATAAGATGAATGCTAAGGATTATTTAATGGCTAAATTAAAAAAAG<br>ATCAATATCCACATTTAGAATTACTTAATGAGTTAGGTCAATATACACTTGAAG<br>CATTAATTGTTTATGTAATCTGTATTTTATATCGCTCTGAATCAACGATGGTTCGT<br>GTTTCAACATTAATTGATCACCTGGATCGACATGTTAAATCACATGTTTTACTAG<br>TTAATCATAAATATCAAATTATTTTAGAAAAAAAAGAAGATGATAAAAATGAG<br>AGTTTATTAACTAATCACCATATTGGTGTAGGATTAGTTGAATTTCTTGTTGAAA<br>GGGATCTAATGGTATTAGAAAATACAGATAATATAAATGTTCCTATTCAAAAG<br>AAGAAGGGTAAATATTTTTTACCTAAACATCTTTTTGCTATCTGTAATTTTGATG<br>TATCATTACTACCAATAAAATTTAATTTACCTATGGTATGTGAACCGTTACCTTG<br>GGGTAGTGCTTTATCAGACAATTTAATCCCTAAATATTTGTCTGACTTAAGAGG<br>AGGTTACTTAAGTGGAATAACTGGAGCAATTAGTCGTTACAATCTGTTAAGCAC<br>GGGGGATATCAATCACTACTATATTGATATTAGTCATAATTATCATACTCTATGT<br>GATGTGATGAACAAACTTCAGAATCAGCCCTTCAAAATATCAAAACATATGTT<br>AACTTTTATAAAAAAATATAAAGAAATGTTTGTTAACTTCGGTTTACTTATGCC<br>AGAGCCT

AmH218/0.015	1061	CACAATGATTGAAACTCCATGTGGGGCCTCGATGCGAGGCAGGTAAGTGCTTG<br>GTTGTCCAACATTTTTCAACTTGCGATTGATAATGGTGTTTCCAGTAAACTCAGG<br>AACATATATAGAGGGATAATTTACATCTAATGCGCTGAAATTGCGAGGACAAT<br>CATATGCATGTTGCTTACTCAACTTTTCCAATATTGTGTTGTTATGATTAATTGCA<br>CAAAGGAGATTAAGATAATCATATTCATTCATGTCATAAACTAAACCAGGATC<br>CATCGCTAGATTAGGTCGTATGTGACCCGAACCATATGCCAAAGGACTCGCTTC<br>ACTCACTCCATCATTATCACGTATCGGCATTCCCTTCTTGTCTAAGGGGCTTGCT<br>GCAAATTATATATTAAACATTAACTCATCTAATTATTTTCTTAAAAAAATACCC<br>CCCACATCCCTATATTTTTCTTTTTAATCAGTCCTATAAAATCCGATACATTTCTA<br>TTTTAGGATATGACTCACAATCTTTTTTAACTGTCATCCACACATTTAGTATGGG<br>GCGTGACCATCGATTAGCATGCCTATCGCCCCAAGCCTTACATAGTTTAGAATT<br>CAAATTGATAATGTATGATTGAGCAATTCAAAAGTTTTAAATTAAAACTGTTTTT<br>ATATACATTAACTTGGACATGGAACTGGAAGAAATCAATTAACCCAATTTCGA<br>TTCCAAATTGTTGAAGTTTTTAAATAGCATATTCTTGTGAAACTATAATAAACCA<br>TAAAAAGAAAATTGAGTACATTAATAACATAGAATTAAAGAAAAAATAGTAT<br>GGGATAGTTTATCAAAATTGAGTTACAATTATTATACAAGATGATTAATTATAA<br>AAATAAAATTATATCAAAAAAATCTAGATTTTCTTTGGCTTCAAAATAATAGGA<br>GTCCTAACAACATGTTTAGCATCTGACCAAACTAAACTTCCAAAAATATAATCA<br>GAAGAAAAATAATTGAGATTAGCTATTAAAGTAAGTTTAAATGGTAATTGTTG<br>ATTGTGTTCAGAAAATACTAGTGTGTTGGGCTC
AmH6/0.57	5312	CCATTGAGAATATGGCCCCGGCAAATGAAATCATCATTTTCCCATTTGCTTCTTT<br>TCCTTGTTTGTTCAAGGGTTTCATCTTCCGATTCCGCCGGCTTAGGGGTACTTAT<br>CACGTAATTAACGCTCAAAGTAGTGAGGAGGAAAAGCATCTTCTTTTGCCAAC<br>GTCGAAAATCGACACCGACAAACTTTTCCAACTTAGCAAAGTTAGATGTAAAA<br>TCTTTGATAGTTGTCATCCCGAAGATAATATCACAAAAGATTATTAAGAACTTA<br>ATAATGCAAAATACAAATTACAAAAGGGAATTTGTATTTAACCCTTTGTTGATG<br>ACAAAATTGAAATTACAAATAACAAAACTAATTTACTTGAAGATTGCTTTGTAA<br>CGACAACCGTTACCCGGAAGCTTGATACTTGTTGTAGGCGTGTGATCACTTTCC<br>TTTTGTGATATTTCCCCCACAAAGGTACTTGGGTTGATGACCGGAAATTCACAA<br>AGAGATACAAACAACACAATATACTCTTAGTACTTAGAGTATATCACTTAACA<br>ACAATGTGTTTGAAAATTATAAGAATTGGAAGCTCTAAACAAGAGTTAATTACT<br>CTCTTGACAAAGCACAAGCAAATGAGAATAAGAGTAGAGGAATTTCAGAAAG<br>GTGTGTCTTCAACTCATCACCAAGACCCTATTTATACTACAAGAGTATAGCCTA<br>AGGGTCATGGCACAATAATCACCCTTAATGATCATATTGATGATCACACAATCA<br>ATACCATTAATCTATGGCATTCATGAGCATTATTCACCAAAATATTCCTTTGTAA<br>CCTCTAAATTAAGAGTGGAATTAGTATTGGAATCCTATGCAATCAATTCAAGGA<br>TTAAGAAACAACCCAACGGTCACTCCTTTAAACTCCTTTTGGACCTAGTATATT<br>AATACATATTGTCCCACTACTTTAGTAGTACTTAGTGATTATAAACTTAGGATTA<br>TTAGACACTAAATGTTCAACAATCCTCCCCCATTTAGGGTATAATAAGCCTCTC<br>ATTTACAAAACAAAACTTATAATACATTTCATGCATCAACGCTAATATCCCTTC<br>GGATTGAATTAACGCCTAGTACAACACACTTCACAAGTGATCGAGCCAGAATT<br>GATTTCCATACGGATTTGAATCAAATGACCCTTCCAACCACTTGAAGATGAAGT<br>ACACACAAAACAAATAACACAAAGTTATTAACCTGACACATTATAAGGCCGTG<br>TCCGTATCTTATTCATAAGTTTATCATAGTCAGTATGCCAATCTTGACTACTTGA<br>AGCGGCCACACTTCAAACTTATATAGGTAGGTTCTCAATACGTATAAAACATAT<br>CCCCGTGATCCGGATACAACACCAAGAGCTCTTCAACCCTTGATCTTGAGAACT<br>TTCAATGGACGACTACCTCGTTCATCACGGTCCAAAGCAACGATGGGTCATTAA<br>ACTTGTTGCTTACAAAAATCAAAAGGACTTTACCACATTGAATTCAGACACGAT<br>GTTACTCATGCGTGAATTGGGCCTTCCCAAATGATTTTATTGACATAAACCGATT<br>TCAACTGAAGCTTGTTCAATTGAATCCCTACTCATGGCTTTCGTAAACGAATCC<br>GCCACATTGAACTTAGTGTTCACATAGTCAAAAGTTATGACCCCGTTAATGATA<br>AGTCCTTGCACTAGGCTATGCCTAAGAGCAATATGACGAGACTTACCATTATAC<br>ACTTGACTATAAGCTCTACCTAAAGCCGTCATGCAATCCGCATGAATCGCCACC<br>GGTGAAATTGGCTTAGGTAACAAAGGTATCTCAAACATGAGATTCCTCAACCA<br>CTCTGCTTCACTAGATGCAGAAGCAAGAGCTATAAACTCAGCTGACATAGTAG<br>AATCAGCTATACATGTTTGTTTCTTAGAAGACCATGAAATGGCACCTCCCCCAT<br>AAACAAACACCCAACCACTTGTAGAAGAATTATCCTCTTCATTAGTGATCCAAC<br>TAGCATCGGAATAACCTTCTAAAATAGGAGGTTCACCGCTATAAGCAATACCA<br>TAATCCTTGGTACGCAACAAGTATCTTAATACTCTTCTTATCGCCATCCAATGAT

		GGGGACCCGGGTTGCTTGTAAATCTACTAAGTTTACCAACCGCAAAAGCTATGT<br>CTGGCCTAGTACAGGTCATGGCATACATCAAAGATCCTATGACCTTTGAATACT<br>CCAATTGAGAAATTGGTTGACCGGTATGTTTGGCAAATTTCATACCTGGATCCA<br>TGGGAGTAGAGACCGGAGCTTTATCAAGCATACTAAACCGTTTAAGTATCTTCT<br>CAACATAGTGAGATTGACTTAACTTAATACGGTTACCTTGTCTTATGATCTTAAT<br>ACCTAAGATCACATCGGCCTCCCCCATATCCTTCATCTGGAAGGATCTAGACAA<br>AAAATCTTTTGTCTCTTGAACTTGTACTAAACTAGTACCAAAGATGAGCATGTC<br>ATCTACATATAGACAAATGATAACCCCATTTCCTTGGTTATCGAACTTGCTATA<br>AACACACTTATCGGCTTGATTCAATTTGAATCCAAAGGACAAAATCGTTTCATC<br>AAACTTTTGATGCCATTGCTTAGGTGCTTGTTTAAGTCCATATAAGGACTTAACA<br>AGTTTACACACCTTTTGCTCTTGTCCTTTCAAAACAAACCCTTCGGGTTGTTTCA<br>TATAAACTTCCTCATCTAATTCCCCATATAGAAATGCGGTTTTAACATCCATTTG<br>ATGTATTTCTAGATGATAGACCGTAGCTAAAGCTACCAATAACCGAATAGTGGT<br>TATTCTTGCAACTGGTGCATATGTATCGAAATGATCGATACCATGCTTTTGTCTA<br>AAGCCTTGTGCCACTAAACGGGCCTTGAACTTGTCAATAGTACCATCAATCTTC<br>ATTTTCTTTCGAAAAATCCACTTGCAACCTATTGGTTTACAAGTAGGAGGAAGA<br>TCCACTAACACCCAAGTGTTATTACCCATGATGGATTGCATCTCATCATCAATA<br>GCCTCTTTCCAAAAGGCGCTATCATGTGATGCCATAGCATCGCTATAAGTCAAT<br>GGGTCACCTTCCACATTATATAAATATGGAACACTAGAAGATACTTCACTTCTA<br>GTACCTTCAACCAAGTATACTTGGAAGTCCGGTCCAAAGCTTTTAGCTTTCCGTT<br>GTCTCTTACTCCTTTTGGGAATAGGATCAACTAATTCTTGTTCATCTAGTTGTTGA<br>TTTTCATCAACACCTAGAGGTGAACCTTGTTGATTATCATCAACATTTTCATTGG<br>TACAAACTTGTAGATTTCTTTGTCTACTCAATGATGAAAATCTATTTTCATCAAA<br>AATTGCATCTCTTGACTCTATGATCGAATGAATCGACATATAGTCATTTTGTTCT<br>ATAACATAGAACCTATAAGTTTTGCTATGAATAGCATAACCTATAAAAATGCAT<br>TCAATCCCTCGTTCACCGAGTTTCTTTCTTTTGTTACCGGGCAATCTTACAATAG<br>CCCTACAACCCCAAACCTTAAGATAGCTTAAGTTTGGTTTCTTTTTGTACCAAAG<br>TTCATAAGGAGTCTCCTTATTCGTTCTCATTGGAACTCGGTTAAGAATGTGACA<br>AGCCGTCAACATTGCTTCACCCCAAAATCCATCACTCAAACCCGAGTAGGATA<br>ACATGGAATTAACCATCTCTTGTAGAGTACGGTTTTTCCTCTCCGCCACACCATT<br>TGATTGTGGTGCGTATCCGGCGGTTGTTTCATGTATAATTCCCATTGATTTAAAA<br>TAATTTGGATCATAATATTCACCTCCTTTATCGGTTCTTAATCTTTTAAGTTTACT<br>ACCAATTTGTATTTCTACTTCATTTTTGTAAATCTTAAAGTGATCAAGAGCCTCA<br>TCTTTTGTATGTAAAAGATATACATAACAAAACTTGGAAAAATCATCAATAAA<br>AGTTATGACATACTTTTTATTTCCCAATGATGGTGTACTATGAAAGTCACAAAG<br>ATCACTATGGATTAATTCCAAAATTTCGGATTTCCGTTCAACACTTTTAAAAGGT<br>GTTCTTGTGATCTTATTTAACATGCACGTTTTGCAATTTTTAGACATTTTAACACA<br>CATAGGAATTAAATCATGCTTAGACATAAAAGAAACTTTATCAAAATTCACAT<br>GTCCTAATCTAACATGCCACAAATAATGCAAATCATCTTTATCATTCATGTTTGA<br>AACATTACAAATAAAGTTCTTATTTAAATCTAAAGACAATTTAAATAAACTATT<br>ACATAGAAAAGCACGTCCTACAAACAAGTTATTTTTAGAGATAATACACCTAT<br>CGGCTTCAAATACTAATTTGTAGCCCAATCGATTCAAAATAGGACCCGAGACA<br>AGATTTCTACTAATTTCGGGTGCATAATACACATCCCTTAAAACTAAGAGATTC<br>CCCGAAGTAAACAATAGCTCAATTTGTCCTTTGCCTTGGACCATGATAGGTGCG<br>TTGTTTCCCATGAAGAGATGCTCCCCATCAACAACCTTGTGAAAGGTTTTGAAC<br>CATTTTGTAGAATTGCATACATGTCTCGATGCCCCCGAATCGATCCACCAAGAC<br>AAAGAATCATCCTGCACAACACTAAAATTAGTATTCATTGAAGAAAGATTCAA<br>AGAAGAATTTTCATTCAAAATTGCAAGATTACCTTGAGTGGAAGGTCCTTTGCT<br>TGTAGAAGCTTGACCACTCCCCTTTGCCTTCTTTTGCTTGTTCTTGAGAACAAAG<br>CAATTTTTCCTTCGGTGTCCGGGCTTGCCACATTCCCAACATACCTTTTCGGTAT<br>TTGAGACAATGGCCTTCCCCTTTGAAGAATCAATTCTCTTCCTCTTATCTCCGGA<br>ACGGGATGGTTTCCCTTCCGCAACCATGTTGATGGTTCCAACATTAGGATTTTCA<br>TCCTTCCGATTCTCATGAATCCTTATGCTAGACTCAACAACAATATGTTGTCCAA<br>GATCGGAGAGTGTAATCTCCTCTCTTTTATGCTTTAAAGCATGTCTAACATCCCT<br>CCATGAGGGAGGCAACTTGTCTATAATGCTTGAAACAAGAAAAACTTCATCTA<br>TTTCAATTTTATGTAATTTAAGGTTGGCATGCATTCTTTGTAATTCATGAAATTG<br>ATCAATTATAGGGCGATCTTCTACCATCTTATAAGAATTAAAATTACTTACAAG<br>AAACTTTTTGGAACTTGCATCCTCCGCCATGTATTTGTTCTCCAAAGCATCCCAT<br>AATTCCTTAGCGGACTCATGGTATTGGTAGATGTCAAAGAGAGAATCTTGCATT

AmH26/0.24	3949	TTTTGAAGTAGAGTGTGATGCATCAGGGGTAGGTATTGGAGGTGTCCTAACTCA<br>AAACAACAAACCCCTTGCTTATTTAGTGAGAAACTCAATGATGCTAAAAGGA<br>AATATTCTACTTATGATAAAGAGTTTTATGCTATTATTAGATGTTTAGAACATTG<br>GAGACATTATTTGATTGCAAAAGAATTTGTGTTGCATTCTGATCATGAAGCTTTA<br>AAATTCATTCAAAGTCAACACAAACTTCAATCTAGGCATGCTAAATGGGTTGA<br>GTTCCTACAAACCTTTCATTTCACTATTAAACACAAAGCGGGTAAACTAAACAC<br>CGGAGCTGATGCTTTATCTAGGAGATACTTGCTTTTGAGTACTTTGAATACTAAG<br>GTTGTTGGAATGGAATTATTAAAAGAATGCTATATTGATGATGTTGATTTTGGA<br>GAAATATTTGAAAAATGTCATAACAAACCTCAAGGACTTTTTTACATCTTTCAA<br>GGATATCTCTTTCGAGGTAATCAATTATGTGTGCCTAGACATAGTGTTAGGGAG<br>ACCTTAATTAAGGAGTATCATGAGGGAGGACTTGGAGGACATTTTGGGGAAGA<br>AAAGACCATAGCTTTAGTGAGTGAGACCTTTTATTGGCCCAAACTTGCTAAAGA<br>TGTCATTCATATTATTAAGAGGTGTGTGCAATGCCTAAAAGCCAAAGCCCACA<br>AAACGTCTCAAGGTCTTTACCAACCTCTTCCGATACCACAAAACCCATGGGAG<br>GATGTGAGCTTAGATTTCATAACCGGACTTCCTAGAACCTTTAACAATAAGGAT<br>TCTATAATGGTTGTTGTTGATAGGTTTTCTAAGATGGCCCATTTCATTGCTTGTCA<br>TACTACTCATGATGCCGTTAACATTGCTAACCTTTACTTTAAAGAGATAGTAAG<br>ACTACATGGGATAGCAAGAAGTATGGTGAGTGATAGGGATTCAAAGTTTCTTA<br>GTCATTTTTGGCTTACTTTGTGGAAAAAGATGGGAACGAAGCTTAACTTTAGTA<br>GTTCTAGCCATCCCCAAACTGATGGTCAGACCGAAGTAACCAATAGGACCTTA<br>GGAACCTTATTGCGTGCCCTCATTACTAAGAATCCTAAACAATGGGAACAATTG<br>CTACCACATGCTGAATTTGCTTATAATCGAGTTCCTAGCAAGACCACCGGTTTTT<br>CTCCTTTCTTTGTGGTTTATGGCCTTAATCCTTTAACCCCTATAGACCTTACACCT<br>TTTCCTACACCTTTGAAGTTTAGTCATGATGCCGAGTCTAGAGCCAATGAGATT<br>CAAAAAGTGCATAAGCAAGTTGTGGAAAGGATTGAGAAAATGAATGAGAAGG<br>TTAAGGAAAGAGTGGATCAAAAGAGAAAAGAAGTTATTTTCAAAGAAGGTGA<br>CTTAGTATGGATTTACTTGAGAAAGGATAGGTTTCCCACACAAAGGAAATCAA<br>AGTTAAGTGCTAGAATTGATGGACCTTTCGAAATTTTAGAAAAAGTAAATGAC<br>AATGCTTACAAACTAGAGCTACCTAGTAGCTATGGTGTGTCTTCTACTTTCAATG<br>TTGCAGATTTAGTTCCATTCCATGAGGCGGACATGTTACCAAGCTTGAGGTCAA<br>GCTTTTCCAAAGAAGGGGAGGATGATGTAGGATTAACTACATCTAATTCTCATG<br>AGCAAGCCAAGATCAATACCATGATTCATCATAGGGAAGACGCTAGAATTCAA<br>GGTCCAAATGTCAAGTCTTTTAATTTTGTAGTCATGAACTAAGTCTTCTTTAGAT<br>TGAGTCGTTTTATTATTTAAGTCTTTTATTTTATTTCGAGTATTTGAGTCTTTTATG<br>TATTTTTCGTTTTGCAATAAACTAAGTTCTTAGAAATGATTAAGCCAGGTTTAGG<br>ATCAAGCTTAATCATGTCTCATGTTTAGTTAGTTGAGTTGTAATTTTTATTTTTCA<br>TTTGTCGAGTCTTTAAATATAAAATCGCTCAAATGGTTCGAGTATTAGACAAAT<br>GAAACGCAAGAACAAAGTAAGAAATATTCAGCTATGAGAGCATCAAATCATC<br>CGAAAATTGCAAGATAGCAAAAGATACATGGAGAATACTTGGAGAATATTTGC<br>AAGATCACAAATAAAGCCGAATCAAGACAATTACTTTGTTTCCAAAAAAAATC<br>AGATTGCATTGAGGATCAATTGAACATTAATACAAAAACCTTTGAGCAAGTTTG<br>CTTTGTTTTGCAATTTATTTTTAACTTGGATTAGTTAAAGTAATTTGAAGTTCCTA<br>TGCTTTTCATTCTTTTGGCTAAAGATTGAATGTGTTCTTAGGAGCCCTTAGTTTTA<br>ATTCGTGGTGTATCTGTATCGAATTAAACCTTTGGTTCAGCCGTTCTTAGTTGAT<br>CTTTTTCAACATATCCCTTTTAATTTTTCTTGTTACAAAACCCACAAAAATCACA<br>AAAACAATTCGATTGAAATCAAAACAGCAAGTAAACGTGTGCATATAGTTTGG<br>TTATTGTTCGAGACGTAGCCAACCTATATCATCTTGGTACCAGAGCCAAGGCTA<br>CGCTCCTAGCCTATATCATTTTTTTCGGTGTGTTCATAGCCCCAAAGATTCATTC<br>AACCGAGCCTAGAAGTCCACAAACGAAAACAAGAATTCTTCACGTTGGCAGTT<br>TCTGTCCCAAACTTCGACAGGCCATAACTTGCTCGTCCGATATCCAAATTACAA<br>AATTCTTTTTTTCATTTTGGGTATTTTTTCGAGATCTATCTTCCTGTAAATTTTCAC<br>AATTTTATGATTTTATTTGCTATTAAACTGGGCAGTTTTTTCGAAAGTATGCAAA<br>CTTGATTGTTTTTGATAATATGGCTCAACGTGAGAAAATTTTCTCCACAAGATGC<br>ACCATTAAACAAAAAGTGTGTTCTTTAATTATCGATGGAGGAACGTGGACAAA<br>CATTGCATCCCAAACCCTTGTTTCCAAACTACAACTTCAAACCATACCTCATCC<br>ACTACCCCACACCATCAAATACCGTAACCAATGTCTACTAGTATCACAAGTAG<br>TTCTACTCCATTTTTCAATTGGTAATAGTTATAAGGAAAGTCTTTTATGTGATGTT<br>ATACCTATGGATGCATGTCATATTCTATTAGGAAGACCTTGGCAATATGATAGA<br>AAAGTTATGCATGATGGCTTTAAAAATACACATACTTTTGTTTTAAATAATAAG

		AAAATCACATTAGCTCCTTATACACCAGCCCATAATTCTAAATTGCAAGCAACTTTAACAAATTTTCATTCTTTGATTCCTATACTTAAAAGTGAACAACATGAATTTATGGAACGAAAAGAATTGTTATTGTTGAATGAAGAAACCGAATCTGTGTTGCATGATCACCCTTTGCTTGTGCCTTTGCTTGATGAATTTGCATATGTTTTTCCTACTGAAATACCAAATGGACTTCCACCTAAAAGGGACATACAACATAAAATTGATTTCATTCCTGGTTCCACGTTGCCTAACAAACCAGCCTATAGAACTAATCCGAAAGAGACCGAAGAAATTAGGAGACAAGTTGATGATTTGTTAGCTAAAGGTTTGATTAGAGAATCCATAAGTCCTTGTGCCGTTCCTACCTTACTTGTGCCTAAGAAAAATGGAGAAATGAGGATGTGTGTGGACAGTAGAGCCATTAATAGAATCACCATCAAGTATAGATTCCCAATACCTAGACTTAATGACTTGTTAGATGATTTATATGGTTCAAAAGTTTTCTCTAAGATTGACCTTAGGAGTGGATATCACCAAGTTAGGATTCATGAAGGGGATGAATGGAAAACCGCATTTAAAACTAAGGAAGGACTTTATGAATGGTTAGTTATGCCCTTTGGTTTATCTAATGCACCATCTACCTTTATGAGACTTATGAACCAGACCTTAAAACCTTTCTTGGGTCAATTTGTTGTGGTTTATTTTGATGATAT

Supplementary Table S14. The tandem DNA repeats identified in genomes of *Amaranthus cruentus*, *Amaranthus hypochondriacus*, and *Amaranthus tricolor,* demonstrating the sequence similarity according to BLAST results (identity/coverage).

Subject / Query	*Amaranthus tricolor*	AmT1	AmT2	AmT129	*Amaranthus cruentus*	AmC4	AmC9	AmC27	AmC32	AmC12	*Amaranthus hypochondriacus*	AmH4	AmH51	AmH9	AmH27	AmH209	AmH3	AmH6	AmH26
Amaranthus tricolor	Size, bp	169	42	683		169	42	42	4950	3008		42	169	169	4133	565	5302	5312	3949
AmT1	169			100%/81%		96%/91%							100%/84%	100%/89%					
AmT2	42						67%/96%	86%/92%					60%/96%						
AmT129	683	95%/81%				85%/75%							49%/72%	92%/78%					
Amaranthus cruentus																			
AmC4	169	96%/91%		100%/77%									99%/85%	100%/98%					
AmC9	42		67%/96%					100%/93%				93%/100%							
AmC27	42		86%/92%				100%/93%					100%/91%							
AmC217	470																21%/74%		
AmC246	271															26%/84%			
AmC32	4950														83%/97%			13%/73%	
AmC12	3008																		41%/93%
Amaranthus hypochondriacu																			
AmH4	42		60%/96%				100%/100%	100%/91%											
AmH51	169	100%/85%		100%/73%		99%/85%								98%/85%					
AmH9	169	100%/90%		98%/77%		100%/98%							98%/85%						
AmH27	4133								99%/96%									13%/72%	
AmH6	5312								12%/73%						10%/71%				
AmH26	3949									32.5%/93%									

Figure S1. Images of *Amaranthus cruentus* var. Dyuimovochka. Chromosomes after (A) FISH with 45S rDNA (green) and 5S rDNA (red) and (C) FISH with 45S rDNA (green) and satDNA AmC9 (red) without α-monobromonaphthalene treatment. Chromosomes after (B) FISH with 45S rDNA (green) and 5S rDNA (red) and (D) FISH with 45S rDNA (green) and satDNA AmC9 (red) with α-monobromonaphthalene treatment. DAPI staining – blue. Scale bar – 5μm.

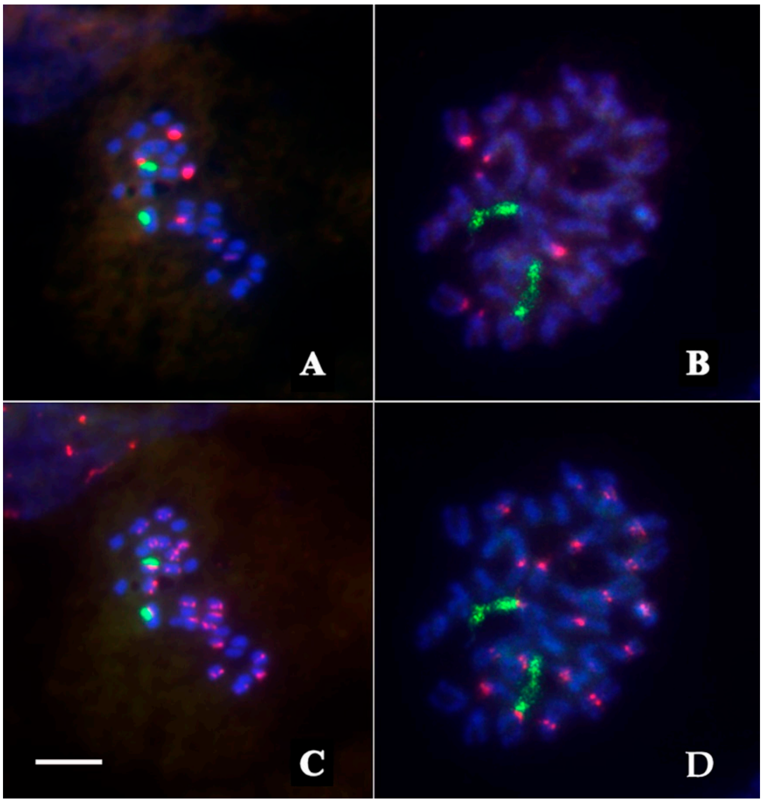

Supplement: Supplementary file 1 [file ijms-26-11026-s001.zip › ijms-3950272-supplementary.pdf]
